# The Enhanced Efficacy of Intracellular Delivery of Doxorubicin/C6-Ceramide Combination Mediated by the F3 Peptide/Nucleolin System Is Supported by the Downregulation of the PI3K/Akt Pathway

**DOI:** 10.3390/cancers13123052

**Published:** 2021-06-18

**Authors:** Ana F. Cruz, Mariana B. Caleiras, Nuno A. Fonseca, Nélio Gonçalves, Vera M. Mendes, Susana F. Sampaio, Vera Moura, Joana B. Melo, Ramiro D. Almeida, Bruno Manadas, Sérgio Simões, João N. Moreira

**Affiliations:** 1 CNC—Center for Neurosciences and Cell Biology, Center for Innovative Biomedicine and Biotechnology (CIBB), University of Coimbra, Faculty of Medicine (Polo 1), Rua Larga, 3004-504 Coimbra, Portugal; ana.filipa.cruz@cnc.uc.pt (A.F.C.); mcaleiras@cnc.uc.pt (M.B.C.); nuno.fonseca@cnc.uc.pt (N.A.F.); nelio.goncalves@cnc.uc.pt (N.G.); vmendes@cnc.uc.pt (V.M.M.); susana.lipa@gmail.com (S.F.S.); vdantasmoura@gmail.com (V.M.); ramirodalmeida@gmail.com (R.D.A.); bmanadas@cnc.uc.pt (B.M.); ssimoes@ci.uc.pt (S.S.); 2 Univ Coimbra—University of Coimbra, CIBB, Faculty of Pharmacy, Pólo das Ciências da Saúde, Azinhaga de Santa Comba, 3000-548 Coimbra, Portugal; 3 TREAT U, SA—Parque Industrial de Taveiro, Lote 44, 3045-508 Coimbra, Portugal; 4 Univ Coimbra—University of Coimbra, CIBB, Institute for Interdisciplinary Research (IIIUC), 3030-789 Coimbra, Portugal; 5 iCBR—Coimbra Institute for Clinical and Biomedical Research, CIBB, Center of Investigation on Environment Genetics and Oncobiology (CIMAGO), Pólo das Ciências da Saúde, Azinhaga de Santa Comba, 3000-548 Coimbra, Portugal; mmelo@fmed.uc.pt; 6 Univ Coimbra—University of Coimbra, Clinical Academic Center of Coimbra (CACC), Faculty of Medicine, Pólo das Ciências da Saúde, Azinhaga de Santa Comba, 3000-548 Coimbra, Portugal

**Keywords:** nucleolin, ovarian cancer, doxorubicin, C6-ceramide, synergistic combination, phospho-Akt downregulation

## Abstract

**Simple Summary:**

Targeted nanomedicine-based approaches that aim at the simultaneous delivery of synergistic drug combinations to multiple cellular populations are of high relevance for tackling heterogeneity on solid tumors. Considering that cancer stem cells (CSC) may originate from non-stem cancer cells, single-drug regimens targeting only one of these cell populations could enable tumors to evade treatments. As such, the identification of a common marker, such as nucleolin, might result in a therapeutic advantage. The results herein generated suggested a transversal role of nucleolin in the internalization of F3 peptide-targeted pegylated pH-sensitive liposomes into bulk ovarian cancer cells, including putative CSC-enriched ovarian cells. The intracellular delivery of a drug combination such as the one tested herein was relevant in the context of cell lines with higher intrinsic resistances to doxorubicin. The enhanced efficacy of the F3 peptide-targeted liposomal combination of doxorubicin/C6-ceramide was supported by the downregulation of the Akt pathway, within a specific range of basal level of expression.

**Abstract:**

Targeting multiple cellular populations is of high therapeutic relevance for the tackling of solid tumors heterogeneity. Herein, the ability of pegylated and pH-sensitive liposomes, functionalized with the nucleolin-binding F3 peptide and containing doxorubicin (DXR)/C6-ceramide synergistic combination, to target, in vitro, ovarian cancer, including ovarian cancer stem cells (CSC), was assessed. The underlying molecular mechanism of action of the nucleolin-mediated intracellular delivery of C6-ceramide to cancer cells was also explored. The assessment of overexpression of surface nucleolin expression by flow cytometry was critical to dissipate differences identified by Western blot in membrane/cytoplasm of SKOV-3, OVCAR-3 and TOV-112D ovarian cancer cell lines. The former was in line with the significant extent of uptake into (bulk) ovarian cancer cells, relative to non-targeted and non-specific counterparts. This pattern of uptake was recapitulated with putative CSC-enriched ovarian SKOV-3 and OVCAR-3 sub-population (EpCAM^high^/CD44^high^). Co-encapsulation of DXR:C6-ceramide into F3 peptide-targeted liposomes improved cytotoxic activity relative to liposomes containing DXR alone, in an extent that depended on the intrinsic resistance to DXR and on the incubation time. The enhanced cytotoxicity of the targeted combination was mechanistically supported by the downregulation of PI3K/Akt pathway by C6-ceramide, only among the nucleolin-overexpressing cancer cells presenting a basal p-Akt/total Akt ratio lower than 1.

## 1. Introduction

Cancer is one of the leading causes of death worldwide and responsible for an estimated 10 million deaths in 2020. Among them, female breast and ovary represented 6.9% and 2.1%, respectively, of the total of cancer deaths in 2020 [1]. The mortality of epithelial ovarian cancer is strongly associated with the asymptomatic nature of this disease, leading to 75% of patients being diagnosed at an advanced stage [2].

The late-stage diagnosis, often accompanied by the development of resistance to conventional chemotherapy and/or tumor recurrence, contributes to the high mortality rate of cancer [3]. Importantly, there is evidence that a putative population of tumor cells, named cancer stem cells (CSC), is involved not only in drug resistance and tumor recurrence, but also in tumorigenicity and metastasization [4,5,6]. A given cell population, selected by any given marker(s), is considered to have CSC phenotype upon presenting a tumorigenic potential (established in vivo) higher than other cell sub-populations [7]. Several surface markers, including CD44, CD24, and metabolic markers, such as aldehyde dehydrogenase, have been successfully used to identify highly tumorigenic putative CSC in breast tumors [8,9]. CD44 and CD24, along with epithelial cell adhesion molecule (EpCAM), have been successfully used to identify putative CSC population in ovarian tumors [10,11].

Furthermore, the PI3K/Akt/mTOR pathway has also been implicated in carcinogenesis, pluripotency and drug resistance through regulation of proliferative and anti-apoptotic mechanisms. Activation of Akt triggers a cascade of responses regulating many normal cellular processes, ranging from cell growth and proliferation to motility and survival but it is often abnormally activated in different tumors [12,13,14]. Moreover, this pathway has been described as essential for CSC proliferation and survival [15,16,17].

Critically, as CSC may originate from non-stem cancer cells, single-drug regimens targeting only one of these cell populations could enable tumors to evade treatments, potentially undermining the therapeutic outcome [18,19]. In this respect, the identification of a common marker, such as nucleolin, can offer an important therapeutic advantage [20,21].

Nucleolin is an abundant protein identified in the nucleolus [22]. It is implicated in DNA and RNA metabolism, controlling chromatin structure, rDNA transcription, rRNA maturation and ribosome biogenesis, as well as in the regulation of cell cycle and transcription [23,24]. Nucleolin expression has been demonstrated at the cell surface of tumor cells of diverse histological origin. In fact, the presence of nucleolin was verified on the cell surface of human lung cancer cell lines and in the cell membrane of human pulmonary carcinoma tissues [25], as well as on the surface of breast cancer stem cells, non-stem breast cancer cells and endothelial cells from tumor blood vessels, rendering it as an important target in cancer therapy in the context of solid tumors [20,26,27]. Targeted nanomedicine-based approaches aiming at simultaneous spatial and temporal delivery of synergistic drug combinations tackling multiple cellular populations could be highly relevant, providing the accessibility to the CSC niche [20,28].

Previously, a pH-sensitive pegylated lipid-based nanoparticle containing a synergistic drug combination of C6-ceramide (pro-apoptotic sphingolipid described to inhibit PI3K/AKT pathway [29,30,31,32]) and doxorubicin (DXR), a cornerstone topoisomerase II inhibitor, and functionalized with the nucleolin-binding F3 peptide, was developed [33]. Accordingly, in the present work, we aimed at unraveling the underlying molecular mechanism of action of the nucleolin-mediated intracellular delivery of C6-ceramide to cancer cells and understand the ability of the F3 peptide-targeted DXR/C6-ceramide combination to target ovarian cancer cells, including ovarian CSC.

## 2. Materials and Methods

### 2.1. Materials

Doxorubicin hydrochloride (DXR) was obtained from MicroBiopharm (Tokyo, Japan). Calcein, 4-(2-hydroxyethyl)piperazine-1-ethanesulfonic acid (HEPES), 2-(*N*-morpholino)ethanesulfonic acid (MES), disodium ethylenediaminetetraacetate dehydrate (EDTA), Trizma^®^Base, resazurin, sephadex G-50, ammonium sulphate, sodium chloride, 3β-hydroxy-5-cholestene-3-hemisuccinate (CHEMS) and cholesterol (CHOL) were purchased from Sigma-Aldrich (St. Louis, MO, USA). The lipids 2-dioleoyl-sn-glycero-3-phosphoethanolamine (DOPE), 1,2-distearoyl-sn-glycero-3-phosphocholine (DSPC), 1,2-distearoyl-snglycero-3-phosphoethanolamine-*N*-[methoxy(polyethyleneglycol)-2000] (DSPE-PEG_2k_), 1,2-distearoyl-sn-glycero-3-phosphoethanolamine-*N*-[maleimide(polyethylene glycol)-2000] (DSPE-PEG_2k_-maleimide), L-α-phosphatidylethanolamine-*N*-(lissamine rhodamine B sulfonyl) (RhoD-PE), *N*-hexanoyl-D-erythrosphingosine (C6-ceramide) were acquired from Avanti Polar Lipids (Alabaster, AL, USA) or Lipoid (Ludwigshafen, Germany). F3 (KDEPQRRSARLSAKPAPPKPEPKPKKAPAKK) and the nonspecific (NS) peptide were custom synthesized by Genecust (Boynes, France). All other chemicals were of analytical grade purity.

### 2.2. Cell Culture

The ovarian SKOV-3 (ATCC^®^ HTB-77™) cancer cell line was cultured in DMEM high glucose (Sigma-Aldrich, St. Louis, MO, USA). The ovarian cancer cell line TOV-112D (ATCC^®^ CRL-11731™), the triple-negative breast cancer cell line MDA-MB-231 (ATCC^®^ HTB-26™), the A549 (ATCC^®^ CCL-185™) and H1975 (ATCC^®^ CRL-5908™) human lung cancer cell lines and the nucleolin-overexpressing cell line MDA-MB-435S (ATCC^®^ HTB-129™) were cultured in RPMI 1640 (Sigma-Aldrich, St. Louis, MO, USA), both supplemented with 10% (*v/v*) of heat-inactivated Fetal Bovine Serum (FBS) (Invitrogen, part of Thermo Fisher Scientific, Waltham, MA, USA), 100 U/mL penicillin, 100 µg/mL streptomycin (Sigma-Aldrich, St. Louis, MO, USA). The OVCAR-3 (ATCC^®^ HTB-161™) ovarian cancer cell line was cultured in RPMI 1640 (Sigma-Aldrich, St. Louis, MO, USA), supplemented with 20% (*v/v*) of heat-inactivated Fetal Bovine Serum (FBS) (Invitrogen, part of Thermo Fisher Scientific, Waltham, MA, USA), 100 U/mL penicillin, 100 mg/mL streptomycin (Sigma-Aldrich, St. Louis, MO, USA). MCF12A (ATCC^®^ CRL-10782™) cell line was cultured in RPMI 1640 (Sigma, St. Louis, MO, USA) supplemented with 5% (*v/v*) of heat inactivated FBS (Invitrogen, part of Thermo Fisher Scientific, Waltham, MA, USA), 100 U/mL penicillin, 100 μg/mL streptomycin (Sigma, St. Louis, MO, USA), 20 ng/mL human Epidermal Growth Factor (Sigma, St. Louis, MO, USA) and 0.5 µg/mL hydrocortisone (Sigma, St. Louis, MO, USA). All cell lines were maintained at 37 °C in a 5% CO_2_ atmosphere up to 1 month (from the original batch) to prevent unwanted mutations. Cells were routinely tested for mycoplasma contamination, following the Center for Neuroscience and Cell Biology (CNC) internal rules, and morphology was assessed by microscopy. Cell lines authentication was performed by short Tandem Repeat Profiling.

### 2.3. Preparation of Liposomes

F3 peptide-targeted liposomes, with or without ceramide, were composed of DOPE:CHEMS:DSPC:CHOL:DSPE-PEG_2k_:DSPE-PEG_2k_-F3: C6-ceramide at 2.66:1.34:2:2:0.62:0.18:2 (18 mol% of ceramide) or 4:2:2:2:0.62:0.18:0 molar ratio, respectively, as previously described [33]. The corresponding non-targeted counterparts were composed of DOPE:CHEMS:DSPC:CHOL:DSPE-PEG_2k_:C6-ceramide at 2.66:1.34:2:2:0.8:2 or 4:2:2:2:0.8:0 [27,33]. Liposomes containing DXR were prepared by the ethanol injection procedure as previously described [20]. Briefly, ethanolic lipid mixtures were added to 300 mM ammonium sulfate solution (pH 6.0) at 60 °C and the resulting liposomes were extruded through stacked polycarbonate membranes (100 nm pore size) using a LiposoFast Basic mini extruder (Avestin, Ottawa, ON, Canada). The buffer was exchanged in a Sephadex G-50 gel column (Sigma-Aldrich, St. Louis, MO, USA) equilibrated with 25 mM HEPES and 10% (*w/v*) sucrose buffer (pH 6.5). Encapsulation of DXR was carried out through ammonium sulfate gradient methodology, upon incubation with liposomes for 1 h at 60 °C. Non-encapsulated DXR was removed using a Sephadex G-50 gel column equilibrated with 25 mM HEPES, 140 mM NaCl buffer (pH 7.4).

For the preparation of calcein-loaded liposomes, ammonium sulfate buffer was replaced by a 40 mM calcein solution, and the resulting liposomes were extruded as described above. Calcein excess was removed through a Sephadex-G50 column equilibrated with 25 mM HEPES, 140 mM NaCl buffer (pH 7.4).

Additionally, to prepare rhodamine B-labeled liposomes, RhoD-PE lipid was incorporated in the above lipid mixture (1 mol% of total lipid), and the ethanol solution was added to 25 mM HEPES, 140 mM NaCl buffer (pH 7.4). The resulting liposomes were extruded as described above.

### 2.4. Subcellular Fractionation and Immunoblot

Protein extracts were prepared in lysis buffer (50 mM Tris-HCl (pH 7.5), 150 mM NaCl, 5 mM EDTA, 1% (*v/v*) Triton X-100, 0.5% (*w/v*) deoxycholic acid and 0.1% (*w/v*) SDS), supplemented with 1 mM dithiothreitol and a cocktail of protease inhibitors (Complete Mini, Roche, Basel, Switzerland) and phosphatase inhibitors (PhosSTOP, Roche, Basel, Switzerland). After centrifugation at 16,100× *g* for 10 min at 4 °C, protein in the supernatant was quantified with the bicinchoninic acid (BCA) assay kit (Pierce, Thermo Fisher Scientific, Waltham, MA, USA). Cell samples were kept as a whole and nuclear and cytoplasmic fractionations were performed as previously described [34]. The samples were then denatured with denaturating buffer (62.5 mM Tris-HCl (pH 6.8), 10% (*v/v*) glicerol, 2% (*v/v*) SDS, 0.01% (*w/v*) bromophenol blue and 5% (*v/v*) β-mercaptoethanol) and boiled at 95 °C for 5 min. Protein extracts were resolved by SDS-PAGE in 10% polyacrylamide gels. For immunoblot analysis, proteins were transferred onto a PVDF membrane by electroblotting (200 mA, 120 min at 4 °C). Membranes were blocked for 1 h at room temperature in Tris-buffered saline (137 mM NaCl, 20 mM Tris-HCl, pH 7.6) containing 0.1% (*v/v*) Tween-20 and 5% (*w/v*) low-fat milk. Membranes were probed overnight, at 4 °C, with the following primary antibodies: monoclonal Phospho-Akt (Ser473) (#4060) and Akt (pan) (#4691) (at dilutions of 1:1000) obtained from Cell Signaling (Danvers, MA, USA), anti-nucleolin monoclonal antibody (clone EPR7952, 3.4 ng/mL, Abcam, Cambridge, UK) or anti-glucosylceramide synthase monoclonal antibody (clone 1E5, at a dilution of 1:500, Sigma-Aldrich, St. Louis, MO, USA). The mouse anti-GAPDH (clone GA1R, 0.5 μg/mL, Thermo Fisher Scientific, Waltham, MA, USA) or the mouse anti-β-Tubulin I (clone SAP.4G5, at a dilution of 1:10,000, Sigma-Aldrich, St. Louis, MO, USA) and the rabbit anti-LaminB1 (clone D9V6H, 1:500, Cell Signaling, Danvers, MA, USA) were used as loading controls, for the cytoplasmic and nuclear fractions, respectively, followed by 2 h incubation at room temperature with the corresponding alkaline phosphatase-conjugated secondary antibodies (Invitrogen, part of Thermo Fisher Scientific, Waltham, MA, USA). Membranes were scanned with the ChemiDoc^TM^ Touch Imaging System (Bio-Rad, Hercules, CA, USA), and quantified using the Image J software v1.49q under linear exposure conditions.

### 2.5. Quantification of Cell Surface Nucleolin

A quarter of a million cells in Eppendorf tubes, placed on ice, were resuspended in 200 µL of cold phosphate buffer saline with 1% bovine serum albumin (PBS-BSA) and maintained on ice for 15–20 min. Cells were then centrifuged for 3 min at 170× *g* and resuspended and incubated in 100 µL of PBS-BSA containing 16.7 µM of DSPE-PEG-F3 micelles to prime nucleolin clustering at the cell surface [35], and 10 μg/mL of mouse antinucleolin-Alexa^®^488 antibody (clone 364-5, lot GR314254-1, Abcam, Cambridge, UK) or IgG1k isotype control (Affymetrix, Santa Clara, CA, USA), for 1 h at 4 °C. Cells incubated with anti-nucleolin-Alexa^®^488 antibody or IgG1k isotype alone were used as controls. Cells were then washed twice and resuspended in PBS-BSA and transferred into cytometry tubes (BD Biosciences, San Jose, CA, USA) for flow cytometric quantification of nucleolin density at the cell surface in a BD FACSCalibur system (BD Biosciences, San Jose, CA, USA), using the QuantumTM Alexa Fluor^®^ 488 MESF microspheres kit (lot 11488, Bangs Laboratories, Inc., Fishers, IN, USA). Exclusion of non-viable cells with 7-aminoactinomycin D (7-AAD) (Sigma-Aldrich, St. Louis, MO, USA) prevented intracellular analysis of nucleolin.

### 2.6. Cellular Association of F3 Peptide-Targeted Nanoparticles with Ovarian Cancer Cell Lines

For the cellular association studies, 200,000 ovarian cancer cells were incubated with 0.2 or 0.4 mM (total lipid) of rhodamine-labeled F3 peptide-targeted or non-targeted liposomes, or liposomes functionalized with a non-specific peptide for 1 or 4 h, at 4 or 37 °C. Upon washing, rhodamine-associated cell fluorescence was analyzed by flow cytometry in a BD FACSCalibur system (BD Biosciences, San Jose, CA, USA) and a total of 20,000 events were collected.

### 2.7. Cellular Association of F3 Peptide-Targeted Nanoparticles with Putative Ovarian Cancer Stem Cells

Half-million SKOV-3 or OVCAR-3 ovarian cancer cells were incubated with 0.4 mM (total lipid) of calcein-loaded liposomes functionalized with the F3 peptide or a non-specific peptide, for 1 or 4 h, at 37 °C. Non-targeted calcein-loaded liposomes were also used as control. After washing, cells were stained, aiming at identifying putative ovarian cancer stem cells. Briefly, cells were incubated with anti-CD44-PE/Cy5 antibody (rat IM7 clone) (Abcam, Cambridge, UK) and anti-EpCAM-PE antibody [mouse VU-1D9 clone] (Life Technologies, part of Thermo Fisher Scientific, Waltham, MA, USA), or with the corresponding isotypes IgG2b (Biolegend, San Diego, CA, USA) and IgG1 K (eBioscience, part of Thermo Fisher Scientific, Waltham, MA, USA), for 30 min at 4 °C, in PBS-BSA. The cell-associated calcein signal was immediately analyzed by flow cytometry in a BD FACSCalibur system (BD Biosciences, San Jose, CA, USA) and a total of 20,000 events were collected. Appropriate controls were used to assure the correct compensation of fluorescence signals in each channel.

### 2.8. Cytotoxicity of F3 Peptide-Targeted Doxorubicin (DXR):C6-Ceramide Liposomal Synergistic Combinations against Ovarian Cancer and Lung Cancer Cell Lines

The cytotoxic potential of F3 peptide-targeted liposomal combinations of DXR:C6-ceramide against ovarian cancer (SKOV-3, OVCAR-3 and TOV-112D) and non-small cell lung cancer (A549 and H1975) cell lines was assessed. Additionally, the cytotoxicity of free DXR or C6-ceramide against ovarian cancer (SKOV-3, OVCAR-3 and TOV-112D) cell lines was also evaluated. Briefly, cancer cells, adherent to 96-well plates, were incubated with serial dilutions of different liposomal formulations containing DXR or free DXR or free C6-ceramide for 1, 4 or 8 h at 37 °C in an atmosphere of 5% CO_2_. Afterwards, cell culture medium was exchanged for a fresh one and the experiment extended up to 96 (TOV-112D and A549), 120 (SKOV-3 and H1975) or 144 h (OVCAR-3), depending on the cell line used. Cell viability was assessed by the resazurin reduction assay, by monitoring absorbance at 570 nm and 600 nm (background) in a Spectramax Gemini EM (Molecular Devices, San Jose, CA, USA). Cell death was calculated from the formula 100 − ((Test_570–600_ − CtrNeg_570–600_)/(Ctr_570–600_ − CtrNeg_570–600_)) × 100, where Test_570–600_, Ctr_570–600_ and CtrNeg_570–600_ were the corrected absorbance for treated cells, untreated controls and negative control, respectively.

### 2.9. Quantification of Intracellular Doxorubicin and C6-Ceramide by Mass Spectrometry

One million ovarian cancer SKOV-3 and triple-negative breast cancer MDA-MB-231 cells were incubated with previously determined concentration corresponding to the IC_50_ of F3 peptide-targeted liposomal DXR:C6-ceramide combination at a molar ratio of 1:1 ([F3]L-DC_6_11). SKOV-3 cells were incubated with 0.63 or 0.49 µM for 4 and 8 h, respectively, MDA-MB-231 cells were incubated with 25.9 or 6.35 µM for 1 and 4 h, respectively, at 37 °C of F3 peptide-targeted liposomal DXR ([F3]L-D), DXR:C6-ceramide combination at a molar ratio of 1:1 ([F3]L-DC_6_11) and the non-targeted counterpart (L-DC_6_11). DXR and C6-ceramide were quantified after extraction from the cultured cells using 500 µL of 80% of methanol and sonicated in a cup-horn for 2 min with 60% amplitude using cycles of 3 s on and 2 s off. Samples were frozen at −80 °C for 1.5 h and sonication was repeated with the same parameters as the previous step. Each sample (100 µL) was spiked with 10 µL of the internal standard solution (daunorubicin and C8-ceramide) and 10 µL of 50% acetonitrile + 0.1% formic acid followed by centrifugation for 20 min at 14,000× *g*, and the supernatant was collected for LC-MS/MS analysis. Calibration curves were prepared in cell-matrix by spiking 100 µL of blank cells with 10 µL of standard solution and 10 µL of the internal standards (IS) solution. Eight calibration points with concentration ranges of 0.009–0.597 µM for DXR and 0.001–0.075 µM for C6-ceramide were analyzed to generate the calibration curves. The samples were analyzed on an LC Nexera system (Shimadzu, Japan) coupled to a hybrid triple quadrupole/linear ion-trap 4000 QTrap mass spectrometer operated by Analyst 1.6.3 (Sciex, Concord, ON, Canada). The injector was a CTC-xt (PAL System, Zwingen, Switzerland). The chromatographic separation was performed using a Gemini C18 column (50 × 2.0 mm, 3 μm, 110 Å, Phenomenex, Torrance, CA, USA) with a 4 × 2.0 mm C18 guard-column (Phenomenex, USA). The flow rate was set at 250 µL/min and mobile phases A and B were 0.1% formic acid in water and 0.1% formic acid in acetonitrile, respectively. The gradient elution was programmed to: 2–30% of B (0.0–0.3 min), 30–60% of B (0.3–3.5 min), 60–99% of B (3.5–4.0 min), 99% of B (4.0–7.9 min), 99–2% of B (7.9–8.0 min) and 2% of B (8.0–8.2 min). The ionization source (ESI Turbo V, Sciex, Concord, ON, Canada) was operated in the positive mode set to an ion spray voltage of 5500 V, 40 psi for nebulizer gas 1 (GS1), 30 psi for the nebulizer gas 2 (GS2), 30 psi for the curtain gas (CUR), and the temperature was 600 °C. All molecules were analyzed by Multiple Reaction Monitoring (MRM) setting Q1 and Q3 at unit resolution, the entrance potential (EP) at 10 eV, the collision gas (CAD) at 8 psi, and the dwell time was 75 ms (Table S1). The volume of injection was 10 µL for all the samples.

### 2.10. Evaluation of mRNA Levels of AKT1 and S6K1

AKT1 and S6K1 mRNA levels were determined on ovarian cancer SKOV-3, triple-negative breast cancer MDA-MB-231 and non-small cell lung cancer A549 and H1975 cell lines, upon incubation with 2 and 32 µM of C6-ceramide encapsulated in F3 peptide-targeted liposomes, for 4 and 24 h at 37 °C. The nucleolin-overexpressing MDA-MB-435S cancer cell was used as additional control [27]. Upon cell collection, total RNA isolation was performed using the NucleoSpin^®^ RNA II kit (Macherey-Nagel, Düren, Germany). Afterwards, RNA concentration and quality were determined using a NanoDrop 2000 (Thermo Fisher Scientific, Waltham, MA, USA). Samples presenting a 260/280 ratio under 1.9 were discarded. Samples of total RNA were stored at −80 °C until use. cDNA was obtained using the NZY First-Strand cDNA Synthesis kit (NZYtech, Lisbon, Portugal) according to the protocol established by the manufacturer, using a Unocycler Thermal Cycler (VWR, Radnor, PA, USA). Using species-specific pairs of primers, AKT1 and S6K1 gene expression was quantified by qRT-PCR using β-ACTIN or HPRT as housekeeping genes for data normalization. The primers were obtained from a primer bank database (http://pga.mgh.harvard.edu/primerbank/, accessed on 10 July 2016) and acquired from Integrated DNA Technologies (IDTDNA, Coralville, IA, USA) (Table S2). NZYSpeedy qPCR Green Master Mix (NZYtech, Lisbon, Portugal) was used to perform analysis of samples that were run in StepOnePlus Real Time PCR Detection System (Applied Biosystems, Thermo Fisher Scientific, Waltham, MA, USA). The data were analyzed using the StepOne software v2.3 and the mRNA fold change was calculated using the 2^−∆∆Ct^ method.

## 3. Results

### 3.1. Nucleolin Is Present on the Cell Surface of Human Ovarian Cancer Cell Lines

Following previous results obtained in breast cancer [20,33], and in order to validate nucleolin as a therapeutic target in ovarian cancer, its expression was assessed in SKOV-3, TOV-112D and OVCAR-3 human ovarian cancer cell lines. Triple-negative breast cancer MDA-MB-231, nucleolin-overexpressing MDA-MB-435S and non-tumorigenic MCF12A cell lines were used as controls.

**Figure 1:**
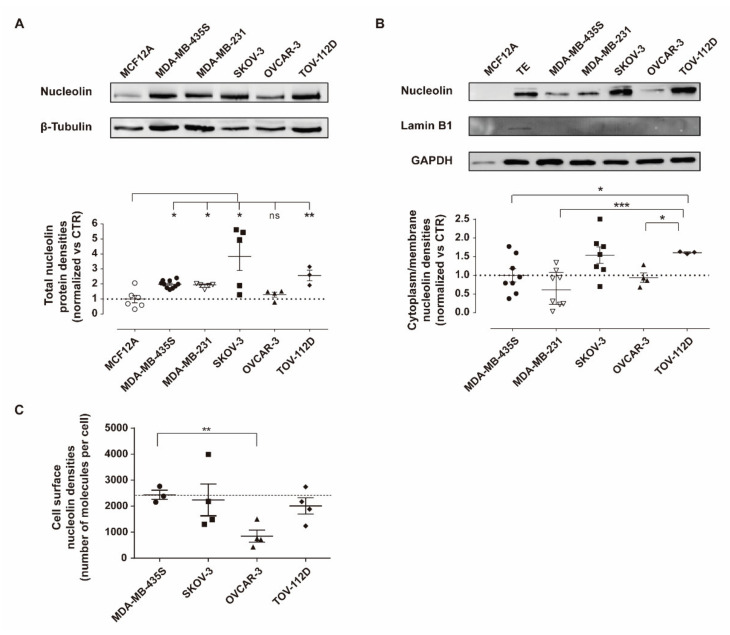
Nucleolin expression density in ovarian cancer cell lines. Quantification of nucleolin protein densities in (**A**) total extracts and (**B**) cytoplasm/membrane extracts was performed by immunoblotting in ovarian cancer cell lines SKOV-3, OVCAR-3 and TOV-112D, as well as triple-negative breast cancer MDA-MB-231, nucleolin-overexpressing MDA-MB-435S and non-tumorigenic MCF12A cells, used as controls. (**C**) Cell surface nucleolin density was quantified by flow cytometry in the indicated cell lines. Data represent mean ± SEM (*n* = 3 unpaired *t* test with Welch’s correction; ^ns^ *p* > 0.05, * *p* < 0.05, ** *p* < 0.01 *** *p* < 0.001). TE = total extract; control (CTR) = β-tubulin or GAPDH. Whole Western Blots images can be found in Figure S5.

SKOV-3 and TOV-112D cells presented 3.8- and 2.6-fold higher total nucleolin levels than the non-tumorigenic cell line MCF12A (Figure 1A; * *p* < 0.05 and ** *p* < 0.01, respectively), and in contrast with the result obtained with OVCAR-3 cells (Figure 1A, *p* > 0.05). In addition, nucleolin was identified in the cytoplasmic/membrane fraction (lamin B1 negative) of all ovarian cancer cells tested (Figure 1B), but not in the one from the non-tumorigenic MCF12A control cell line. The TOV-112D cell line presented a cytoplasmic/membrane nucleolin density that was 1.6- and 2.5-fold higher than in MDA-MB-435S (* *p* < 0.05) and MDA-MB-231 (*** *p* < 0.001) cells, respectively (Figure 1B). Cytoplasmic/membrane nucleolin densities in SKOV-3 and OVCAR-3 cells were similar to triple-negative breast cancer MDA-MB-231 cells and the nucleolin-overexpressing control cell line herein used, MDA-MB-435S. Interestingly, upon assessing the levels of merely surface nucleolin by flow cytometry, some of the previous-mentioned differences were actually dissipated (Figure 1C). Only the OVCAR-3 cell line presented lower levels of cell surface nucleolin than the nucleolin-overexpressing control cell line herein used (** *p* < 0.01), MDA-MB-435S (Figure 1C).

Following the validation of nucleolin presence at the cell surface of ovarian cancer cell lines, cellular association studies were performed in order to confirm the potential of F3 peptide-targeted intracellular drug delivery strategy towards ovarian cancer cells, including ovarian CSC.

### 3.2. Improved Association of F3 Peptide-Targeted Liposomes to Ovarian Cancer Cells

**Figure 2:**
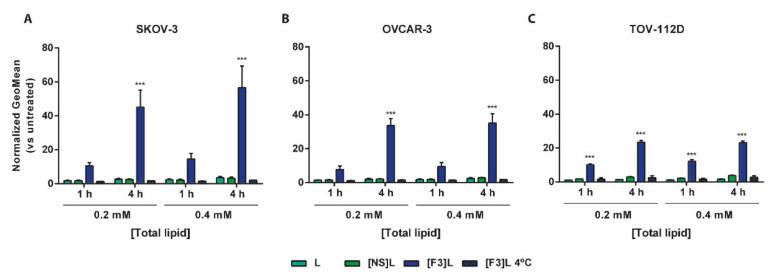
Cellular association of F3 peptide-targeted liposomes by ovarian cancer cell lines. Cells were incubated with 0.2 or 0.4 mM (total lipid) of rhodamine-labelled liposomes functionalized with F3 peptide ([F3]L) or non-specific peptide ([NS]L) or non-targeted (L) for 1 or 4 h, at 37 or 4 °C. The normalized (vs. untreated) rhodamine geometric mean fluorescence for (**A**) SKOV-3, (**B**) OVCAR-3 and (**C**) TOV-112D ovarian cell lines is represented. Data represent mean ± SEM (*n* = 3; two-way ANOVA; *** *p* < 0.001 Bonferroni’s post-test).

Similar to the previous observations with breast cancer cells [27], the F3 peptide-targeted liposomes ([F3]L) presented higher cellular association relative to the non-targeted and non-specific counterparts in ovarian cancer cell lines (Figure 2). Fourteen to sixteen-fold higher association to SKOV-3 (Figure 2A), OVCAR-3 (Figure 2B) and TOV-112D (Figure 2C) ovarian cancer cells, than to the non-targeted (L) counterparts, was observed following 4 h of incubation at the highest concentration tested. At 4 °C, a temperature not permissive to endocytosis [36], a significant decrease of cellular association was observed, thus suggesting that an energy-dependent internalization was taking place, which was in line with previous observation on breast cancer cells [27].

Overall, these results, along with the results obtained in the previous section, suggested the potential of the developed F3 peptide-targeted delivery platform for intracellular drug delivery to ovarian cancer bulk cell lines. It was then addressed the question on whether the observed uptake pattern in bulk ovarian cancer cells could actually be translated to ovarian CSCs.

### 3.3. F3 Peptide-Mediated Intracellular Drug Delivery into Ovarian Cancer Stem Cells

The putative ovarian CSC population was identified based on EpCAM and CD44 expression in SKOV-3 and OVCAR-3 ovarian cancer cell lines, as previously described [10]. Although those markers have been described in a broad sense as ovarian CSC markers [10,11], TOV-112D ovarian cancer cell line in fact lacked the expression of surface CD44, being thus ruled out this set of experiments.

**Figure 3:**
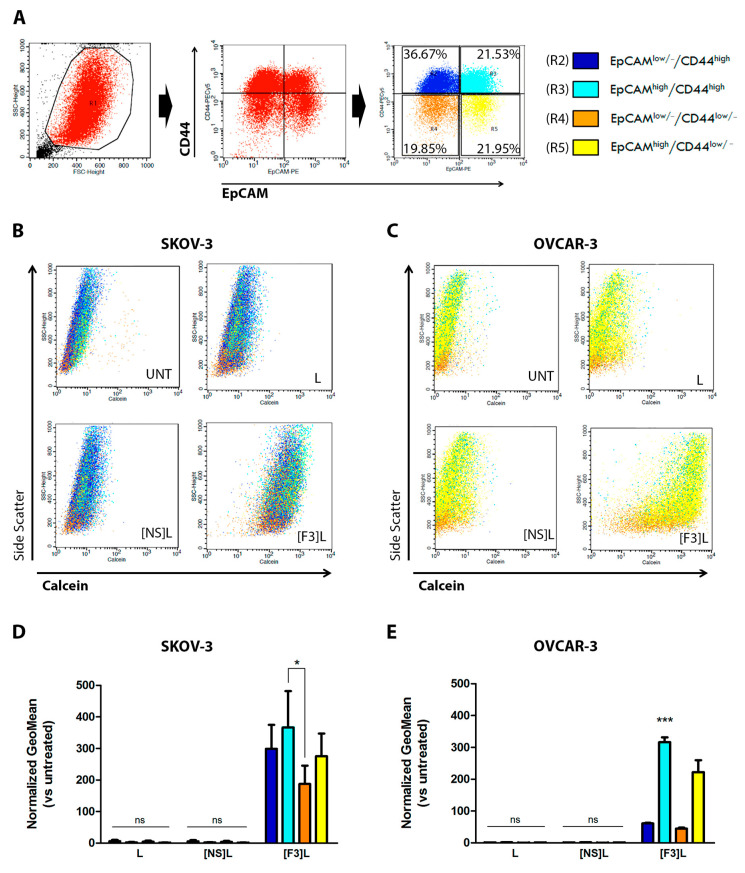
Cellular association of F3 peptide-targeted liposomes with putative ovarian cancer stem cells. (**A**) Flow cytometry gating strategy for the identification of CSC-enriched (R3, light blue) and non-stem cancer cells, -SCC (R4, orange) sub-populations based on the assessment of CD44 and EpCAM surface levels is represented. The calcein/side scatter dot-plots reflecting the calcein signal distribution for (**B**) SKOV-3 and (**C**) OVCAR-3 bulk cells is represented, upon incubation with 0.4 mM (total lipid) of calcein-labeled F3 peptide-targeted ([F3]L), non-specific peptide targeted ([NS]L) or non-targeted (L) liposomes for 4 h at 37 °C. The geometric mean of calcein fluorescence levels for each of the indicated sub-populations for (**D**) SKOV-3 and (**E**) OVCAR-3 cell lines are represented. Data represent the mean ± SEM (*n* = 3; two-way ANOVA with Bonferroni’s post-test; ^ns^ *p* > 0.05; * *p* < 0.05 and *** *p* < 0.001).

CD44 and EpCAM markers, suitable for staining in flow cytometry, enabled the establishment of a gating strategy aiming at identifying not only the putative CSC (EpCAM^high^/CD44^high^) and the non-stem cancer cell (EpCAM^low/−^/CD44^low/−^) populations, but also putative intermediate cell populations (EpCAM^high^/CD44^low/−^ and EpCAM^low/−^/CD44^high^ populations) (Figure 3A). Accordingly, the cellular association of F3 peptide-targeted liposomes ([F3]L) with the different cell sub-populations was assessed. Results clearly indicated that the F3 peptide-targeted liposomes presented higher cellular association relative to the non-targeted and non-specific counterparts in all cell sub-populations studied (Figure 3B,C). This targeting capacity of the former was further reflected into a 2.0- (*p* < 0.05) or 7.1-fold (*p* < 0.001) higher cellular association to EpCAM^high^/CD44^high^ SKOV-3 (Figure 3D) or OVCAR-3 (Figure 3E) sub-populations (CSC) than to the corresponding EpCAM^low/−^/CD44^low/−^ non-stem cancer cell populations (non-SCC). These results supported the capacity of lipid-based nanoparticles functionalized with the nucleolin-binding F3 peptide to target, simultaneously, multiple cancer cell populations, including ovarian CSCs.

To further reinforce the therapeutic potential of the targeted drug combination, the cytotoxicity was then assessed.

### 3.4. F3 Peptide-Targeted Drug Combinations Enabled Higher Cytotoxicity Than Targeted Liposomes Containing Doxorubicin Alone in Ovarian Cancer Cells

**Figure 4:**
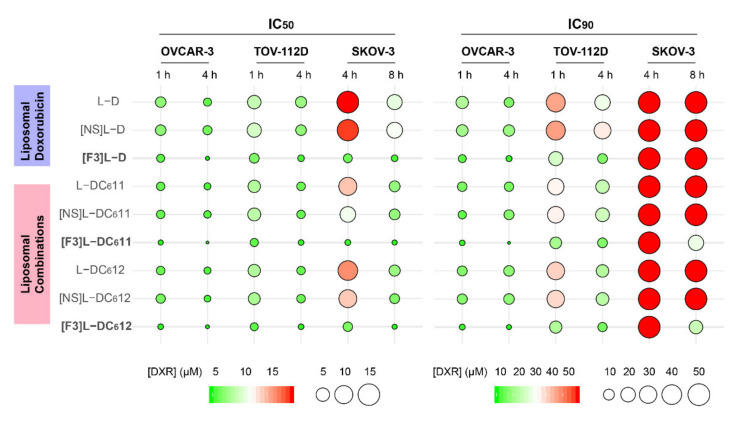
Cytotoxicity of different liposomal combinations of doxorubicin (DXR) and C6-ceramide against ovarian cancer cell lines. The indicated ovarian cancer cell lines were incubated for 1, 4 or 8 h with F3 peptide-targeted liposomal DXR ([F3]L-D) or DXR:C6-ceramide combination at a molar ratio of 1:1 or 1:2 ([F3]L-DC_6_11 and [F3]L-DC_6_12, respectively), at DXR serially diluted concentrations. The experiment was further prolonged for a total of 96 (TOV-112D), 120 (SKOV-3) or 144 h (OVCAR-3) after which cell viability was assessed. Additional controls included liposomes either functionalized by a non-specific peptide ([NS]L-D, [NS]L-DC_6_11 and [NS]L-DC_6_12) or non-targeted (L-D, L-DC_6_11 and L-DC_6_12), incubated under the same experimental conditions. The mean DXR concentrations enabling 50% (IC_50_) or 90% (IC_90_) cytotoxicity are presented, where the circle size and color reflected the mean DXR concentration value (µM, *n* = 3): the smaller the size and the greener the color, the higher the cytotoxic potency.

To evaluate the cytotoxic potential of the targeted drug combinations of DXR:C6-ceramide, the impact of each formulation on the in vitro viability of ovarian cancer cell lines (SKOV-3, OVCAR-3 and TOV-112D) was assessed. Herein, a new presentation strategy was adopted (Figure 4), in order to clearly communicate the determined IC_50_ and IC_90_ values (Table S3) and facilitate analysis. In Figure 4, these values were presented as colored circles, reflecting the mean DXR concentration value: the smaller the size and the greener the color, the higher the cytotoxic potency.

The analysis of the IC_90_ values (Figure 4) clearly evidenced that the SKOV-3 cell line is the most resistant one to the majority of the tested liposomal samples (IC_90_ higher than 50 µM), encapsulating either single (DXR) or drug combinations, targeted or non-targeted, for both 4 or 8 h incubation time. This higher resistance is particularly evident at the IC_90_. In fact, only the targeted drug combinations, either at a 1:1 ([F3]L-DC_6_11) or 1:2 molar ratio ([F3]L-DC_6_12) enabled a measurable 90% of SKOV-3 cancer cells death (22.61 and 16.69 µM, respectively), following an 8 h incubation.

Still within the IC_90_ values, but now moving to the TOV-112D cell line, the samples enabling the greener and smaller circles were still the targeted drug combinations, either at a 1:1 ([F3]L-DC_6_11) or 1:2 molar ratio ([F3]L-DC_6_12), closely followed by the targeted liposomes containing only DXR, for both 1 (11.93, 13.31, and 18.70 µM, respectively) and 4 h (7.73, 6.75, and 7.72 µM, respectively) of incubation. With this cell line, a dependence on the incubation time was evidenced, for all of the tested samples, towards an increase of activity. As we move to the more sensitive cell line (OVCAR-3), differences among the IC_90_ values tend to dissipate. Nevertheless, the lowest values are still associated with [F3]L-DC_6_11 and [F3]L-DC_6_12, for both incubation times: 2.18 and 2.39 µM for 1 h and 1.11 and 1.77 µM for 4 h of incubation, respectively. Similar trends were observed within the set of IC_50_ values (Table S3).

Overall, these results demonstrated that the co-encapsulation of the DXR:C6-ceramide combination within F3 peptide-targeted liposomes improved cytotoxic activity relative to the liposomes containing DXR alone, in an extent that depended on the cancer cell line and on the incubation time.

### 3.5. Intracellular Delivery of Doxorubicin and C6-Ceramide to Cancer Cells Present Different F3 Peptide Dependencies

To unravel some of the mechanisms supporting the improved cytotoxicity of the targeted drug combination, the capability of the developed F3 peptide-targeted lipid-based nanoparticle to enable intracellular drug delivery was assessed upon quantifying intracellular DXR and C6-ceramide by mass spectrometry. This experiment was performed with the triple-negative breast cancer MDA-MB-231 or ovarian cancer SKOV-3 cells, as in both, incubation with targeted drug combinations of DXR and C6-ceramide in a molar ratio of 1:1 enabled a 90% cell death, following 4 or 8 h, respectively, a level of cell death not reached by the counterpart containing DXR alone ([33] and Figure 4, respectively). The experiment was performed at the IC_50_ of the targeted combination for each of these cell lines (at the corresponding incubation times) in order to provide in the end, a cell density enabling drug quantification.

**Figure 5:**
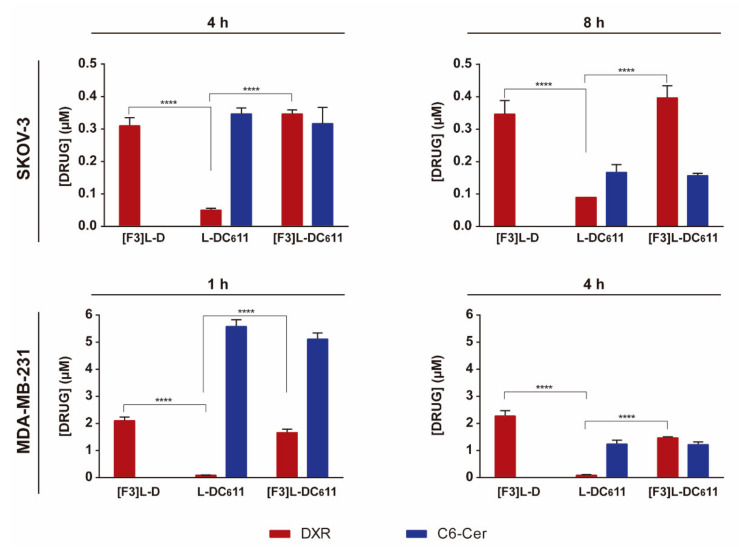
Quantification of intracellular doxorubicin and C6-ceramide by mass spectrometry. Cells were incubated with previously determined DXR concentrations corresponding to the lowest IC_50_ of the [F3]L-DC_6_11 formulation. SKOV-3 cells were incubated with 0.63 and 0.49 µM for 4 and 8 h, respectively, and MDA-MB-231 with 25.87 and 6.35 µM for 1 and 4 h, respectively, at 37 °C with F3 peptide-targeted liposomal DXR ([F3]L-D) or DXR:C6-ceramide combination at a molar ratio of 1:1 ([F3]L-DC_6_11) or the non-targeted counterpart (L-DC_6_11). DXR and C6-ceramide were quantified after extraction from the cultured cells. Data represent the mean ± SEM (*n* = 3; two-way ANOVA with Tukey’s multiple comparisons post-test; **** *p* < 0.0001).

With this set of cells, an interesting observation arose. Regardless of the cell line or incubation time tested, the targeted formulation ([F3]L-DC_6_11) enabled an intracellular accumulation of DXR that was always significantly higher than the one from the non-targeted counterpart (L-DC_6_11), which instead was similar in the case of C6-ceramide (Figure 5), thus suggesting different mechanisms of drug uptake. As expected, the targeted formulation ([F3]L-DC_6_11) enabled intracellular levels of DXR similar to the ones from F3 peptide-targeted liposomes containing only DXR ([F3]L-D) in SKOV-3 and MDA-MB-231 cells.

The mechanistic contribution of C6-ceramide to the improved cytotoxicity of F3 peptide-targeted liposomal combination was then assessed.

### 3.6. Defining the Mechanistic Contribution of C6-Ceramide towards the Improved Cytotoxic Effect of F3 Peptide-Targeted Liposomal Combination

#### 3.6.1. C6-Ceramide-Mediated Improved Cytotoxicity of the F3 Peptide-Targeted Drug Combination Is Supported by the Downregulation of Phosphorylated Akt

Doxorubicin is a well-known topoisomerase II inhibitor that leads to dose-dependent cell death by necrosis, either in its free form or upon delivery by F3 peptide-targeted liposomes [33,37,38]. However, the mechanistic contribution of C6-ceramide to the improved cytotoxicity of F3 peptide-targeted liposomal combination is yet to be defined [33]. Thus, as ceramides have been reported to inhibit the PI3k/Akt pathway [39], it was then questioned whether the mechanism remains the same upon intracellular delivery mediated by the F3 peptide/nucleolin system into cancer cells with different basal levels of phosphorylated/active Akt. Specifically, this protein was evaluated in cells in which F3 peptide-targeted drug combinations enabled higher cytotoxicity relative to targeted liposomes containing DXR alone, namely ovarian cancer SKOV-3 cells (Figure 4 and Table S3), triple-negative breast cancer MDA-MB-231 and nucleolin-overexpressing (positive control) MDA-MB-435S cells [33], all of them characterized by a low intrinsic activation (ratio p-Akt/total Akt lower than 1) of this signaling pathway (Figure S1). This assessment was further extended to cell lines presenting higher levels of basal p-Akt (ratio p-Akt/total Akt equal or higher than 1), as the non-small cell lung cancer (NSCLC) A549 and H1975 cells (Figure S1), described to overexpress nucleolin and to bind and internalize pegylated pH-sensitive liposomes functionalized with the F3 peptide [25].

**Figure 6:**
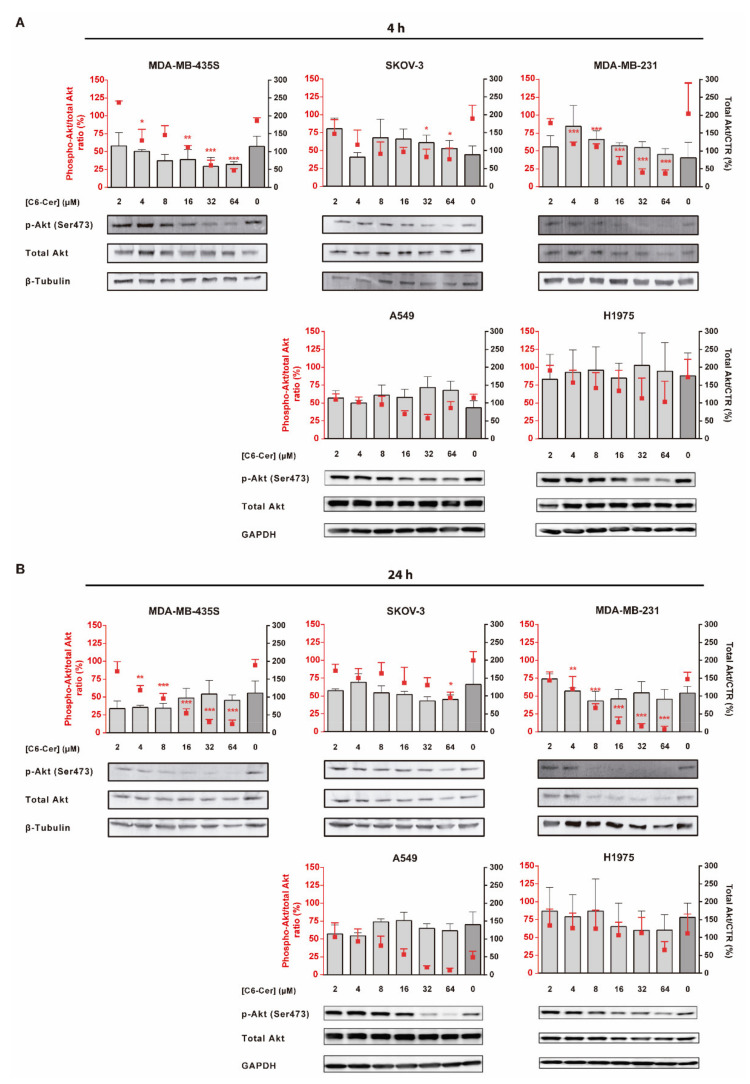
Evaluation of the modulation of p-Akt protein level by F3 peptide-targeted C6-ceramide liposomes in cancer cell lines of diverse histological origin. MDA-MB-435S, SKOV-3, MDA-MB-231, A549 and H1975 cells were incubated with the indicated concentration of C6-ceramide encapsulated in F3 peptide-targeted liposomes ([F3]L-C_6_) for (**A**) 4 h or (**B**) 24 h at 37 °C. Extracted cellular proteins were analyzed by immunoblotting and band signals for p-Akt (Ser473) and Akt were quantified through densitometry imaging, and the p-Akt/total Akt ratio (red squares) and total Akt/Control (bars) for each condition were calculated. Equal amounts of protein were loaded in each lane. Data represent the mean ± SEM (*n* = 3; one-way ANOVA with Dunnett’s post-test; * *p* < 0.05, ** *p* < 0.01 and *** *p* < 0.001). Whole Western Blot images can be found in Figure S5.

On MDA-MB-231 cells, characterized by the lowest levels of p-Akt (Figure S1), a clear dose- and time-dependent decrease of the phosphorylated protein was observed (Figure 6A). The effect became significant at 4 µM F3 peptide-targeted liposomal C6-ceramide onward, following 4 (Figure 6A, *p* < 0.001) or 24 h (Figure 6B, *p* < 0.01) of incubation, relative to untreated cells. At 64 µM, a 5.4- or 25.8-fold decrease on p-Akt levels were observed following 4 (Figure 6A, *p* < 0.001) or 24 h (Figure 6B, *p* < 0.001), respectively, relative to untreated control (Figure 6A,B). A similar dose- and time-dependent trend was observed with MDA-MB-435S (Figure 6A,B), characterized by similar levels of p-Akt (Figure S1).

As the levels of p-Akt doubled, as in the case of SKOV-3 cells (Figure S1), a significant decrease of the phosphorylated protein became significant (*p* < 0.05) only at 32 or 64 µM of F3 peptide-targeted liposomal C6-ceramide (Figure 6A,B). In this case, the previously referred time-dependent effect was not evident.

For cell lines with a basal ratio p-Akt/total Akt equal or higher than 1 (Figure S1), as the A549 or H1975 NSCLC cell lines, respectively, neither the concentrations of F3 peptide-targeted liposomal C6-ceramide tested nor the incubation time enabled a significant downregulation of the levels of p-Akt. A slight decrease in p-Akt levels was observed merely at higher doses, following 24 h incubation (Figure 6A,B). This could in fact justify in the NSCLC cell lines tested, the absence of relevant differences between the cytotoxic activity of F3 peptide-targeted liposomes containing DXR and C6-ceramide combinations ([F3]L-DC_6_11 or [F3]L-DC_6_12) and their counterpart containing only DXR ([F3]L-D]) (Figure S2 and Table S4).

Overall, these results pointed out that C6-Ceramide-mediated improved cytotoxicity of the F3 peptide-targeted drug combination is supported by the downregulation of phosphorylated/active Akt, an effect dependent on the basal levels of p-Akt (elucidated in the graphical abstract).

#### 3.6.2. The Improved Cytotoxicity of the F3 Peptide-Targeted Drug Combination Mediated by C6-Ceramide Relies on a Direct Akt-Mediated Downregulation of the Signaling Pathway

**Figure 7:**
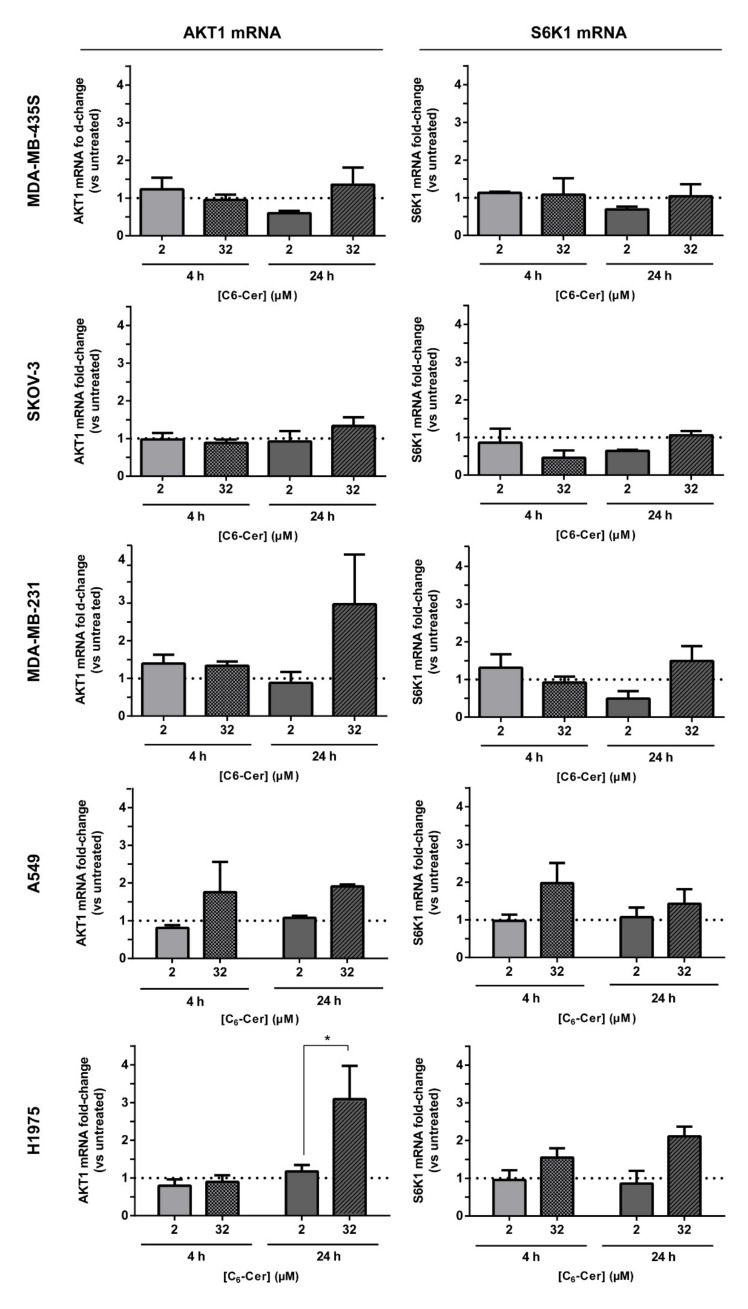
Impact of F3 peptide-targeted liposomal C6-ceramide in the mRNA levels of PI3K/Akt pathway-associated effectors in cell lines of diverse histological origin. Representation of the relative mRNA fold-change of AKT1 and S6K1 in MDA-MB-435S, SKOV-3, MDA-MB-231, A549 and H1975 cells, following incubation with the indicated concentration of C6-ceramide encapsulated in F3 peptide-targeted liposomes ([F3]L-C_6_) for 4 and 24 h, at 37 °C, relative to untreated control. Data represent the mean ± SEM (*n* = 3; *p*-value was calculated using *t*-test, * *p* < 0.05).

To rule out that the decreased levels in p-Akt derived from a reduction in mRNA synthesis, and thus not necessarily to a direct Akt-mediated downregulation of the signaling pathway by C6-ceramide, mRNA levels of Akt and S6K1, a downstream target of the signaling pathway, were evaluated under the same conditions used in the experiments above. In fact, C6-ceramide, delivered by F3 peptide-targeted liposomes, had a minimal impact on the mRNA levels of Akt, except for MDA-MB-231 and H1975 cell lines (Figure 7). In these cells, 24 h incubation with F3 peptide-targeted liposomal C6-ceramide at 32 µM led to a marked increase of mRNA levels of Akt. For MDA-MB-231, at this concentration and incubation time, 80% of cell death was observed [33] and, as Akt is involved in cancer cell survival [12,13], the remaining cells have increased mRNA levels of Akt. In fact, PI3K/Akt/mTOR pathway has been demonstrated to play a critical role in survival and proliferation of the more resistant putative breast cancer stem cell population [17]. The inhibition of this pathway by specific PI3K and mTOR inhibitors (LY294002 and rapamycin, respectively) led to a reduction of cell survival and tumorigenicity of the putative breast cancer stem MCF-7 cell population, defined by the side population assay [17]. A similar trend was observed for NSCLC H1975 cell line. Additionally, C6-ceramide, delivered by F3 peptide-targeted liposomes, had a minimal impact on the mRNA levels of S6K1. Only with MDA-MB-435, SKOV-3 and MDA-MB-231, a decrease of S6K1 mRNA levels were evidenced by at least 1.5-fold relatively to untreated control, following incubation with 2 µM of F3 peptide-targeted liposomal C6-ceramide ([F3]L-C_6_) for 24 h, but without statistical significance (Figure 7).

Overall, these results supported the improved cytotoxicity of the F3 peptide-targeted drug combination upon a direct Akt-mediated downregulation of the signaling pathway by C6-ceramide.

## 4. Discussion

Nucleolin is an abundant protein physiologically present in the nucleus but also in the cytoplasm and at the cell surface in disorders as cancer. Cell surface nucleolin serves as a binding partner of different molecules (as P-selectin, hepatocyte growth factor or pleiotrophin), thus mediating their biological activities, such as cell differentiation, adhesion and leukocyte trafficking, inflammation, angiogenesis and tumor development [40,41]. Nucleolin tumor overexpression has been demonstrated in tumors of diverse histological origin, including in patient-derived samples, as in breast cancer [21] and lung cancer [25]. Gains on therapeutic efficacy, in vivo, arising from nucleolin overexpression has been further evidenced, for example, in pancreatic cancer [42] or mesothelioma [28]. In respect to the latter, our group has unraveled nucleolin as an accessible tumor-associated marker for drug delivery into solid tumors, upon intravenous administration of pegylated pH-sensitive liposomes functionalized with the nucleolin-binding F3 peptide. A significant tumor growth inhibition of orthotopic mesothelioma tumors was observed, paralleled by an impairment of the nucleolin-positive vasculature and downregulation of typically overexpressed genes in patients [28].

Herein, it was demonstrated by Western blot that nucleolin was overexpressed in the cytoplasm and cell membrane (Figure 1B) of cell lines derived from different sub-types of patient-derived ovarian cancer as endometrioid ovarian carcinoma (TOV-112D) and high-grade serous ovarian carcinoma (OVCAR-3 and SKOV-3) [43], similar (excepting TOV-112D, *p* < 0.05) to the nucleolin positive control MDA-MB-435S cells. Interestingly, assessment of cell surface density alone, by flow cytometry, dissipated any previous differences relative to MDA-MB-435S, while evidencing a decrease on OVCAR-3 (*p* < 0.01; Figure 1C). Notwithstanding, no significant differences have been identified among the ovarian cancer cell lines tested (*p* > 0.05), with cell surface levels significantly higher than the non-neoplastic cell line tested, in line with previously demonstrated nucleolin overexpression in ovarian cancer SKOV-3 and OVCAR-3 cell lines [44].

These results (Figure 1C) have been further reflected on an augmented cellular uptake of liposomes functionalized with the nucleolin-binding F3 peptide into the aforementioned (bulk) ovarian cancer cell lines (Figure 2). The extent of uptake was similar among these cell lines, regardless of the time and lipid concentration tested (*p* > 0.05), in line with cell surface nucleolin expression (Figure 1C), and 14- to 16-fold higher than the non-targeted counterparts. A similar range of increased uptake of F3 peptide-(also rhodamine-labeled) targeted liposomes herein used (9.7–17-fold) by nucleolin-overexpressing cell lines had been previously observed, namely in triple-negative breast cancer as the MDA-MB-231 cells [27]. Importantly, the same pattern of uptake was verified with the putative CSC-enriched ovarian SKOV-3 and OVCAR-3 sub-population (EpCAM^high^/CD44^high^) (Figure 3) [10]. These results were in fact in accordance with previously determined 3.2- and 2.6-fold higher cellular association of F3 peptide-targeted liposomes to putative CSCs population (ALDH^high^/CD44^high^) in breast cancer MCF-7 and MDA-MB-231 cell lines, respectively, than to the non-stem cell cancer population (ALDH^low/−^/CD44^low/−^) [20]. This is quite a relevant aspect as CSC are involved in different biological functions of the tumor, including resistance and recurrence [4,6,7,45], being thus quite important to have a system able to target different cell sub-populations in tumors of diverse histological origin.

Surprisingly, however, the pattern of improved uptake of the targeted formulation among the ovarian cancer cell lines was not reflected at the cytotoxicity level. This was particularly relevant at the IC_90_ level of SKOV-3 cells, where the intrinsic resistance to DXR, relative to OVCAR-3 and TOV-112D (Figure S4), supported the absence of activity of that drug alone, regardless of the formulation, targeted or non-targeted, or incubation time. Mechanistically, the lower sensitivity of SKOV-3 cell line to DXR was associated with a p53 gene mutation and a defective activity of the apoptotic protease-activating factor-1 and consequent lack of caspase 9 activation [46,47,48,49].

Often to improve antitumor efficacy and circumvent drug resistance, the therapeutic strategies rely on drug combination approaches. Among others, the combination of conventional chemotherapeutic drugs, such as paclitaxel, sorafenib or gemcitabine with liposomal C6-ceramide (acting at the level of PI3K/Akt/mTOR, essential to CSC proliferation and survival [29]), presented superior efficacy relative to monotherapies in ovarian [50], breast [51] and pancreatic [52] cancer mouse models. In this respect, it is important to synchronize the pharmacokinetics of individual drugs, aiming at the accumulation of a synergistic ratio at the tumor site. In fact, drug co-encapsulation into liposomal carriers has demonstrated to fulfill this need [53]. This was the rationale underlying the development of the liposomal formulation encapsulating defined ratios of C6-ceramide and DXR, where the functionalization with the nucleolin-binding F3 peptide, and subsequent intracellular delivery, enabled synergistic cell cytotoxicity against the triple-negative breast cancer cells [33]. A side-by-side comparison on the intracellular drug levels herein performed for the first time, to the best of our knowledge, supported the importance of dual drug delivery (Figure 5). F3 peptide-targeted formulations enabled similar levels of intracellular DXR into SKOV-3 cells, after 4 and 8 h (Figure 5), but only with the presence of intracellular C6-ceramide (at 8 h of incubation), a measurable IC_90_ value of 16.69 or 22.61 µM, for [F3]L-DC_6_12 or [F3]L-DC_6_11, respectively, was achievable (Figure 4, Table S3).

Interestingly, similar intracellular levels of C6-ceramide in ovarian cancer SKOV-3 and triple-negative breast cancer MDA-MB-231 cell lines, upon delivery by non-targeted and targeted liposomes, suggested a passive delivery process, in contrast to DXR. This was actually in line with the previously described cellular uptake of short-chain sphingolipids [54]. In fact, these (as C6-ceramide) are associated with a rapid transbilayer diffusion upon contacting with other phospholipid bilayers [55,56]. Accordingly, Khazanov et al. [54] demonstrated that the extent of uptake of liposomes, based on the uptake of radiolabeled ^3^H-DPPC, by C-26 cells was significantly lower than that of radiolabeled ^14^C-C6-ceramide at all times tested, suggesting the latter being translocated into the cells in its free form. Importantly, the extent of the transbilayer diffusion associated with short-chain sphingolipids was reduced in the presence of DSPE-PEG_2k_ in the same formulation [54]. Moreover, intravenous administration of liposomal C6-ceramide has shown tumor growth inhibition of a syngeneic mouse solid tumor model of mammary adenocarcinoma [31] and human hepatocellular carcinoma xenografts [57], relative to liposomes without C6-ceramide or untreated controls, further reinforcing the potential of C6-ceramide as an anticancer drug.

Mechanistically, the cytotoxic activity may be underlined by two sets of mechanisms. While DXR is a topoisomerase II inhibitor inducing cell necrosis even when actively delivered [33,37], the activity of intracellular delivered C6-ceramide was suggested to be dependent on the intrinsic activation of PI3K/Akt signaling pathway (Figures S1 and S2). Downregulation of p-Akt took place only in cell lines in which the F3 peptide-targeted liposomal formulation encapsulating defined ratios of C6-ceramide and DXR enabled an increase in cytotoxicity (Figure 4 and Figure 6). The higher basal levels of p-Akt in lung cancer cell lines, and associated similar cytotoxicity between the targeted drug combination and a targeted formulation containing DXR alone, suggested the requirement of a higher dose of the sphingolipid than the one delivered, to enable an advantage of the former formulation. Therefore, basal levels of phosphorylation of Akt may predict whether F3 peptide-targeted liposomal combination enable an improved impact on cell viability (graphical abstract), over the counterpart containing DXR alone. Accordingly, Lin et al. had previously observed that hyperactivation of Akt by a long non-coding RNA led to resistance to Akt inhibitors in triple-negative breast cancer MDA-MB-231 cell line [58].

Additionally, basal protein levels of glucosylceramide synthase (GCS), an enzyme involved in ceramide metabolism [59] has been associated with underlying drug resistance [60,61]. High protein GCS levels in SKOV-3, A549 and H1975 (equal or above 0.4), relative to MDA-MB-231 and MDA-MB-435S (Figure S3), could actually limit the ceramide-induced downregulation of Akt phosphorylation (Figure 6). These results were actually in line with the previously reported high GCS protein expression and the associated less susceptibility to vinorelbine-induced apoptosis in A549 cells [61].

These observations were accompanied by a minimal impact on the mRNA levels of AKT1 and S6K1 (Figure 7), a downstream target of PI3K/Akt/mTOR pathway, suggesting a direct Akt-mediated downregulation of the signaling pathway by C6-ceramide, as a mechanism supporting the improved cytotoxicity of the F3 peptide-targeted drug combination. Akt protein is characterized by three different isoforms, but only Akt1 has been strongly associated with cell survival and growth, namely within cancer. Growth retardation and increased spontaneous apoptosis in the testes and thymus were in fact observed in Akt1 knockout mice relative to wild-type [12,62], whereas increased phosphorylation levels of Akt1 have been described in cells grown under sphere conditions (an in vitro indicator of stemness) [16].

Importantly, data suggested that pathway inhibition is not due to Akt degradation but rather to its deactivation. This is consistent with the proposed interaction of ceramide with PI3KC2β, an upstream effector of Akt pathway, suppressing its activation, and consequently decreasing p-Akt levels in ovarian cancer cells [63]. Moreover, the decrease in p-Akt levels has been associated with the reduction of the putative CSC population [16,64]. He et al. demonstrated that PI3K/Akt knockdown or pharmacological inhibition reduced the putative CSC population, whereas PI3K/Akt overactivation (via PTEN inhibition) led to CSC expansion in MCF-7 breast cancer and J82 bladder cancer cell lines [65]. Moreover, VS-5584, a selective dual inhibitor of mTORC1/2 and class I PI3K, preferentially abolished CSC and delayed tumor regrowth in small-cell lung cancer xenograft models after cessation of treatment with cisplatin [66]. These observations, along with the results demonstrating the cellular association of F3 peptide-targeted liposomes into putative CSC-enriched ovarian population (Figure 3), supported the hypothesis that this formulation, encapsulating defined ratios of C6-ceramide and DXR, could be effective against putative CSC-enriched population. Additional research on this topic will be the focus of our future work.

## 5. Conclusions

Overall, upon a certain level of surface nucleolin expression as determined by flow cytometry, the results herein generated pointed towards a transversal role of nucleolin in the internalization of F3 peptide-targeted pegylated pH-sensitive liposomes into bulk ovarian cancer cells, including putative CSC-enriched ovarian SKOV-3 and OVCAR-3 cells (EpCAM^high/^CD44^high^). Interestingly, while the active intracellular delivery of DXR has been reinforced, C6-ceramide uptake seemed to be associated with a passive process, independent from the presence of the F3 peptide. Intracellular delivery of a drug combination as the one herein tested revealed to be relevant in the context of cell lines with higher intrinsic resistance to DXR, as in the case of ovarian SKOV-3 cells. In fact, the enhanced efficacy of the F3 peptide-targeted liposomal combination of DXR/C6-ceramide was supported by the downregulation of the Akt pathway, within a specific range of basal level of expression.

## Data Availability

Data is contained within the article or supplementary material.

## Supplementary Materials

The following are available online at https://www.mdpi.com/article/10.3390/cancers13123052/s1, Figure S1: Intrinsic activation of PI3K/Akt signaling pathway in cell lines of diverse histological origin, Figure S2: Cytotoxic activity of doxorubicin (DXR) and C6-ceramide combinations encapsulated in different liposomal formulations against NSCLC cell lines, Figure S3: Basal protein levels of glucosylceramide synthase (GCS) in cell lines of diverse histological origin, Figure S4: Cytotoxic potential of free doxorubicin (DXR) and C6-ceramide (C6-Cer) against ovarian cancer cell lines, Figure S5: Whole Western Blots, Table S1: Multiple Reaction Monitoring (MRM) transitions of the data acquisition method for doxorubicin, C6-ceramide, daunorubicin, and C8-ceramide, Table S2: List of primer nucleotide sequences for qRT-PCR, Table S3: Cytotoxicity of different liposomal DXR:C6-ceramide combinations against ovarian cancer cell lines, Table S4: Cytotoxicity of different liposomal DXR:C6-ceramide combinations against non-small cell lung cancer cell lines.
