# Supplementary material for: The Enhanced Efficacy of Intracellular Delivery of Doxorubicin/C6-Ceramide Combination Mediated by the F3 Peptide/Nucleolin System Is Supported by the Downregulation of the PI3K/Akt Pathway
Source: Cancers (Basel). 2021 Jun 18;13(12):3052. doi: 10.3390/cancers13123052 (PMC8235382; doi:10.3390/cancers13123052)
Supplement: Supplementary file 1 [file cancers-13-03052-s001.zip › cancers-1249567-supplementary.pdf]

Supplementary Materials: The Enhanced Efficacy of Intracellular Delivery of Doxorubicin/C6-Ceramide Combination Mediated by the F3 Peptide/Nucleolin System is Supported by the Downregulation of the PI3K/Akt Pathway

Ana F. Cruz, Mariana B. Caleiras, Nuno A. Fonseca, Nélio Gonçalves, Vera M. Mendes, Susana F. Sampaio, Vera Moura, Joana B. Melo, Ramiro D. Almeida, Bruno Manadas, Sérgio Simões and João N. Moreira

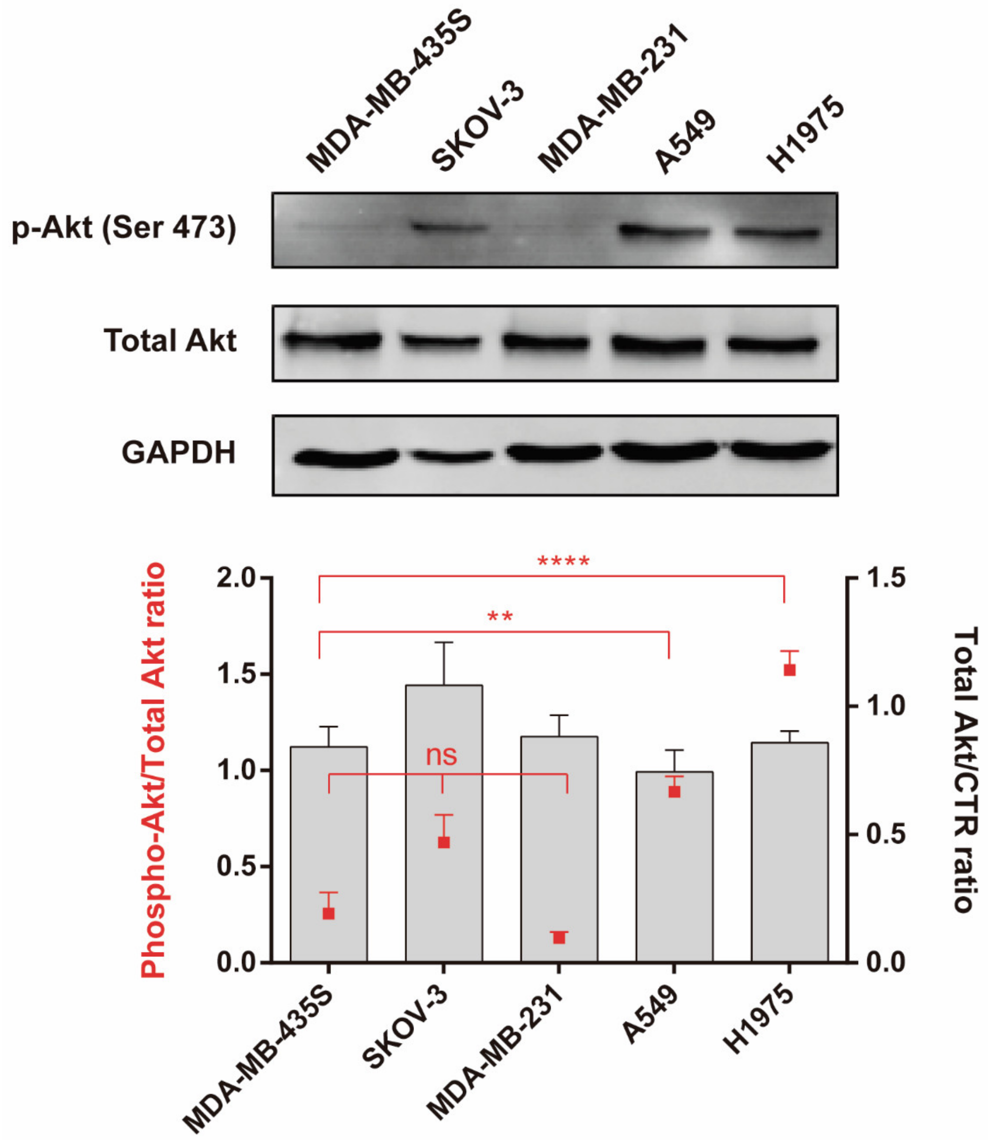

Figure S1. Intrinsic activation of PI3K/Akt signaling pathway in cell lines of diverse histological origin. Total protein extracts of ovarian cancer SKOV-3, triple-negative breast cancer MDA-MB-231, NSCLC H1975 and A549 and nucleolin-overexpressing MDA-MB-435S cell lines were analyzed by immunoblotting. Band signals for p-Akt (Ser473), Akt and Control were quantified by densitometry imaging. P-Akt/total Akt (squares) and total Akt/GAPDH (bars) ratios were calculated for each cell line. Equal amounts of protein were loaded in each lane. Data represent the mean ± SEM (n = 3; 2-Way ANOVA with Tukey's multiple comparisons post-test; ** $p < 0.01$, **** $p < 0.0001$, ^{ns} $p > 0.05$).

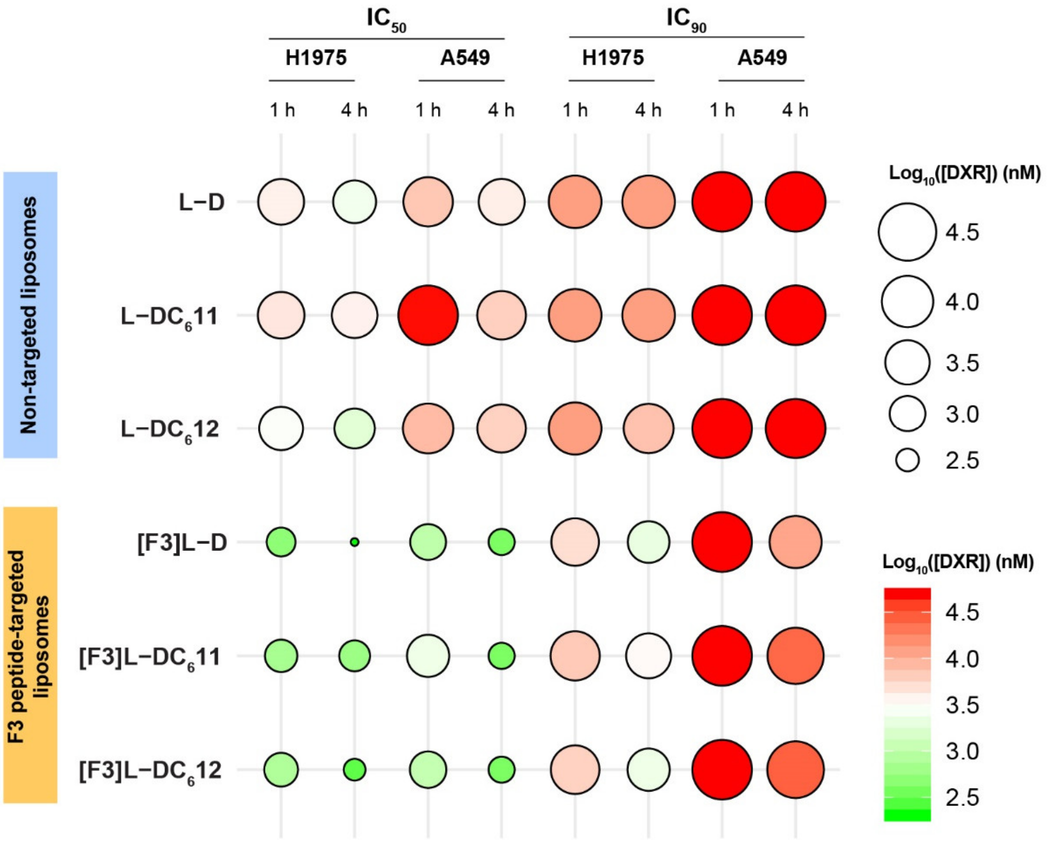

Figure S2. Cytotoxic activity of doxorubicin (DXR) and C6-ceramide combinations encapsulated in different liposomal formulations against NSCLC cell lines. H1975 and A549 NSCLC cells were incubated for 1 or 4 h with F3 peptide-targeted liposomal doxorubicin, DXR ([F3]L-D) or DXR:C6-ceramide combination at a molar ratio of 1:1 or 1:2 ([F3]L-DC611 and [F3]L-DC612, respectively), at DXR serially diluted concentrations. The experiment was further prolonged for total of 96 (A549) or 120 h (H1975), after which cell death was assessed by the resazurin reduction assay. Results were compared with non-targeted liposomes (L-D, L-DC611 and L-DC612), incubated under the same experimental conditions. The mean DXR concentrations enabling 50% (IC_{50}) or 90% (IC_{90}) cytotoxicity are presented, where circle size and color reflected the mean DXR concentration value (nM, log_{10} scaling, *n* = 3): the smaller size and the greener the color, the higher the cytotoxic potency.

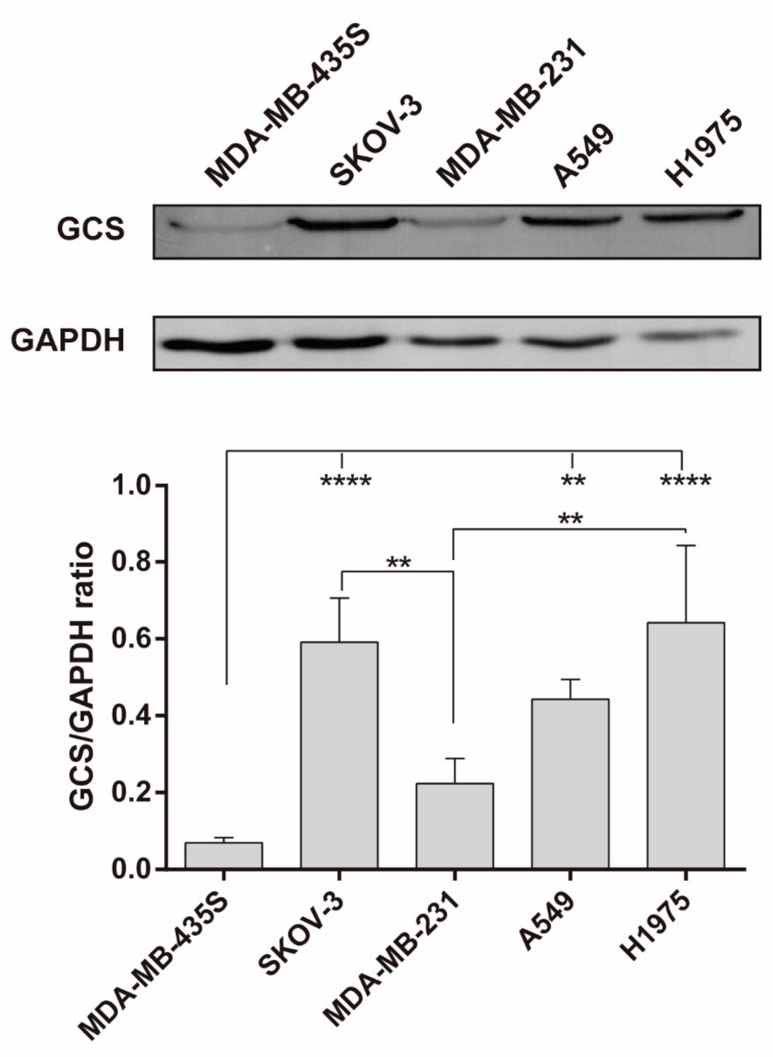

Figure S3. Basal protein levels of glucosylceramide synthase (GCS) in cell lines of diverse histological origin. Total protein extracts of ovarian cancer SKOV-3, triple-negative breast cancer MDA-MB-231, NSCLC H1975 and A549, and nucleolin-overexpressing MDA-MB-435S cell lines were analyzed by immunoblotting. Band signals for GCS and control were quantified by densitometry imaging. Total GCS/GAPDH ratios were calculated for each cell line. Equal amounts of protein were loaded in each lane. Data represent the mean ± SEM ($n = 3$; One-Way ANOVA with Tukey's multiple comparisons post-test; ** $p < 0.01$, **** $p < 0.0001$).

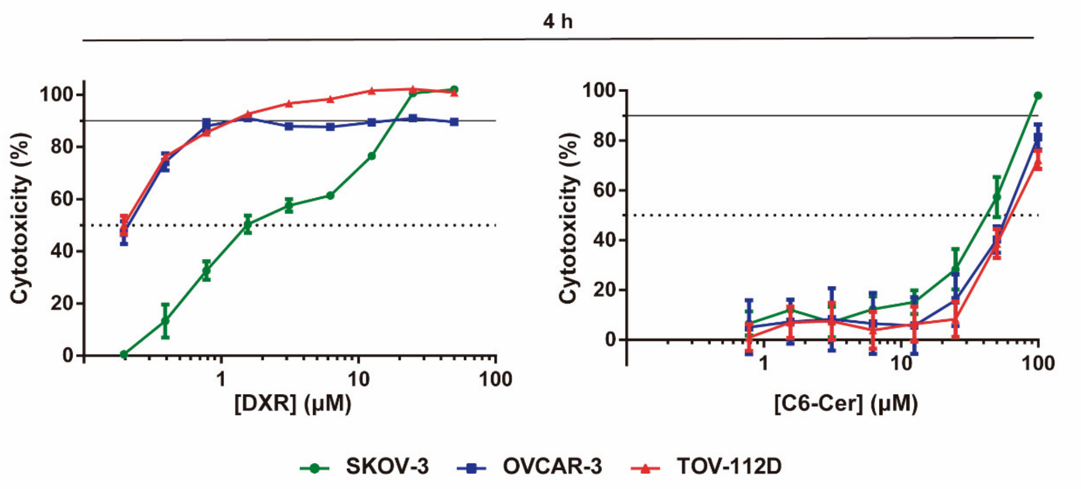

Figure S4. Cytotoxic potential of free doxorubicin (DXR) and C6-ceramide (C6-Cer) against ovarian cancer cell lines. SKOV-3, OVCAR-3 and TOV-112D human ovarian cancer cells were incubated for 4 h with serially diluted free DXR or C6-Ceramide. The experiment was further prolonged for a total of 96 (TOV-112D), 120 (SKOV-3) or 144 h (OVCAR-3) and cytotoxicity was assessed by the resazurin reduction assay. Dose-response curves were then generated, and data points represent the mean ± SD, for each concentration tested (doted and full lines represent 50 and 90% cytotoxicity, respectively).

Figura S1 - Basal p-Akt

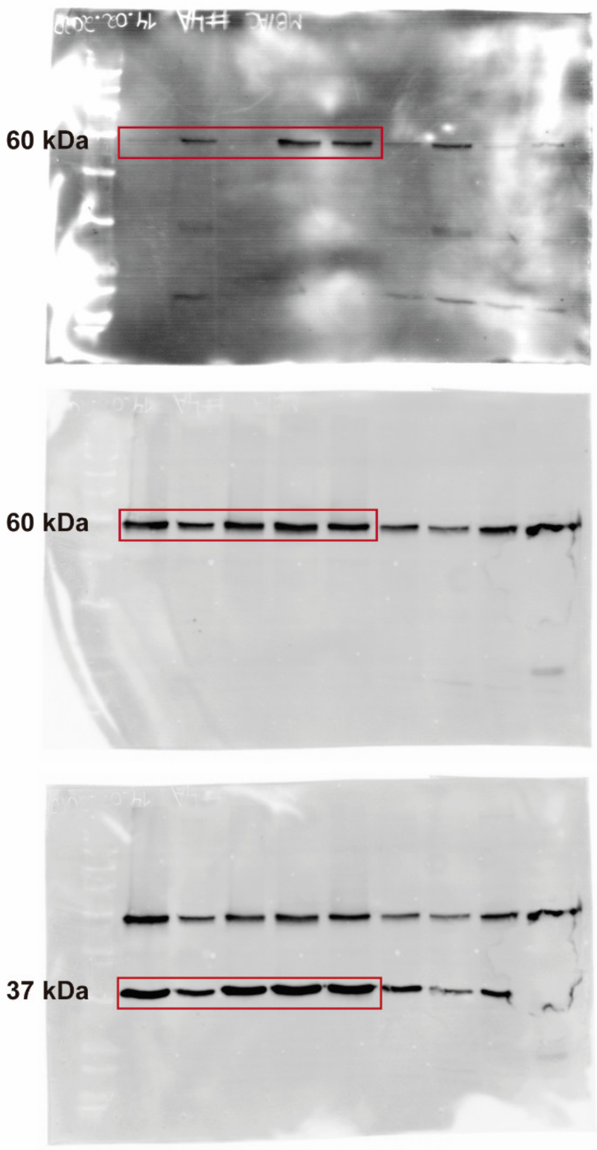

Figura 1B - Nucleolin cytoplasm/membrane extracts

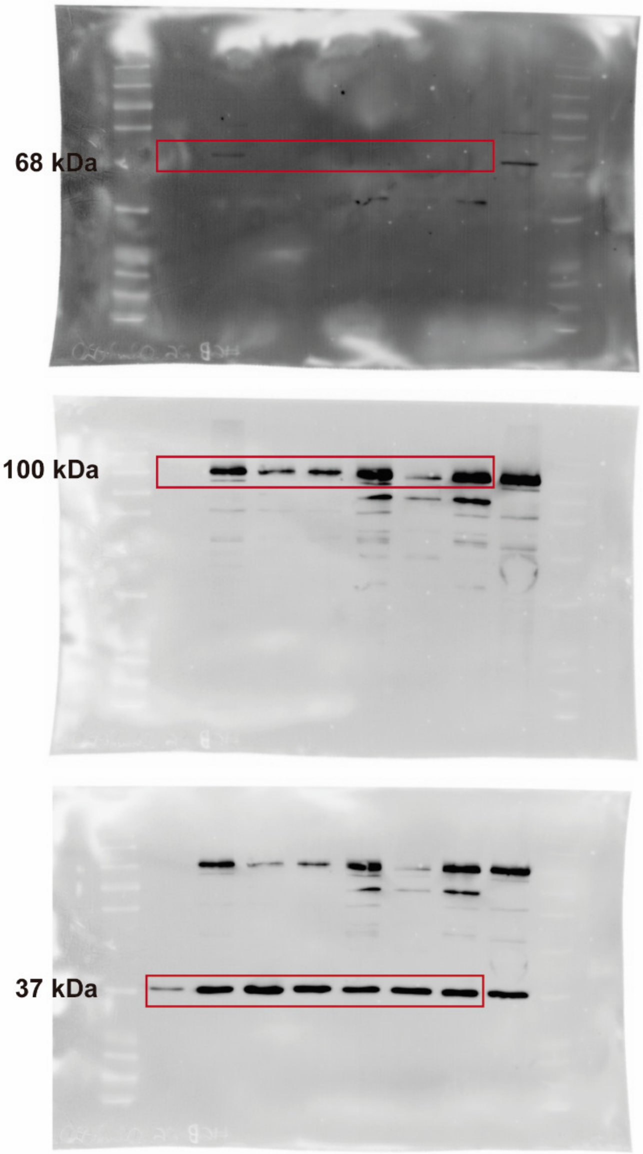

Figura 1A - Nucleolin total extracts

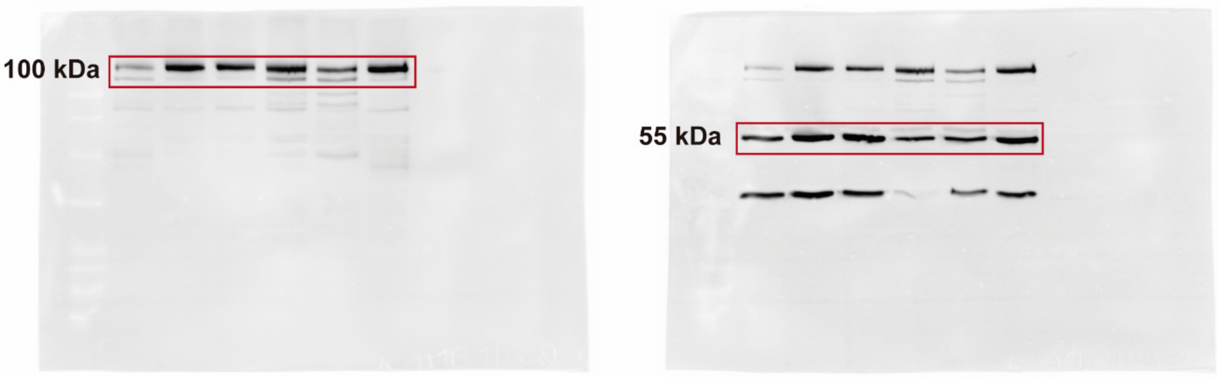

Figura 6 - p-Akt/total Akt ratio

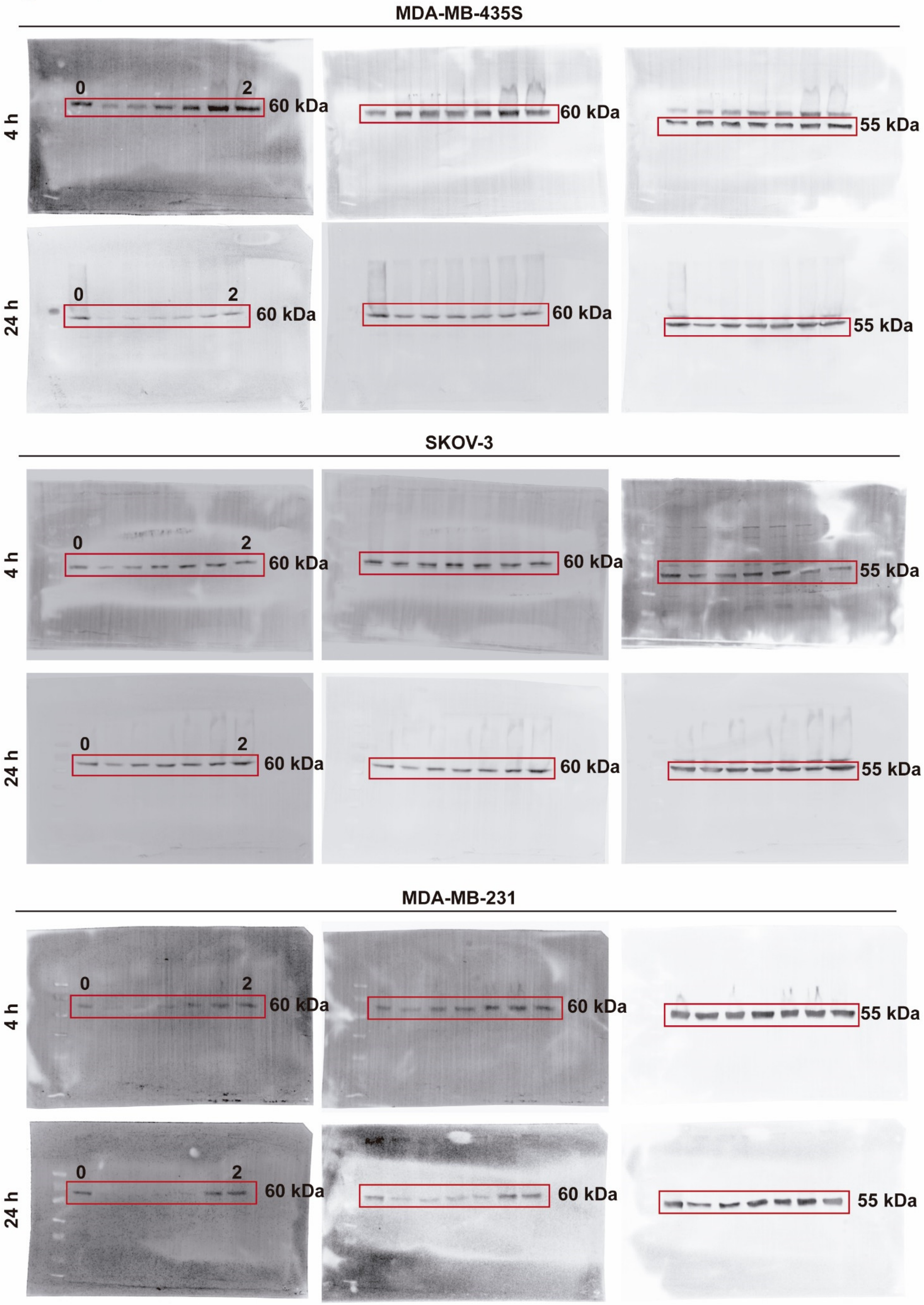

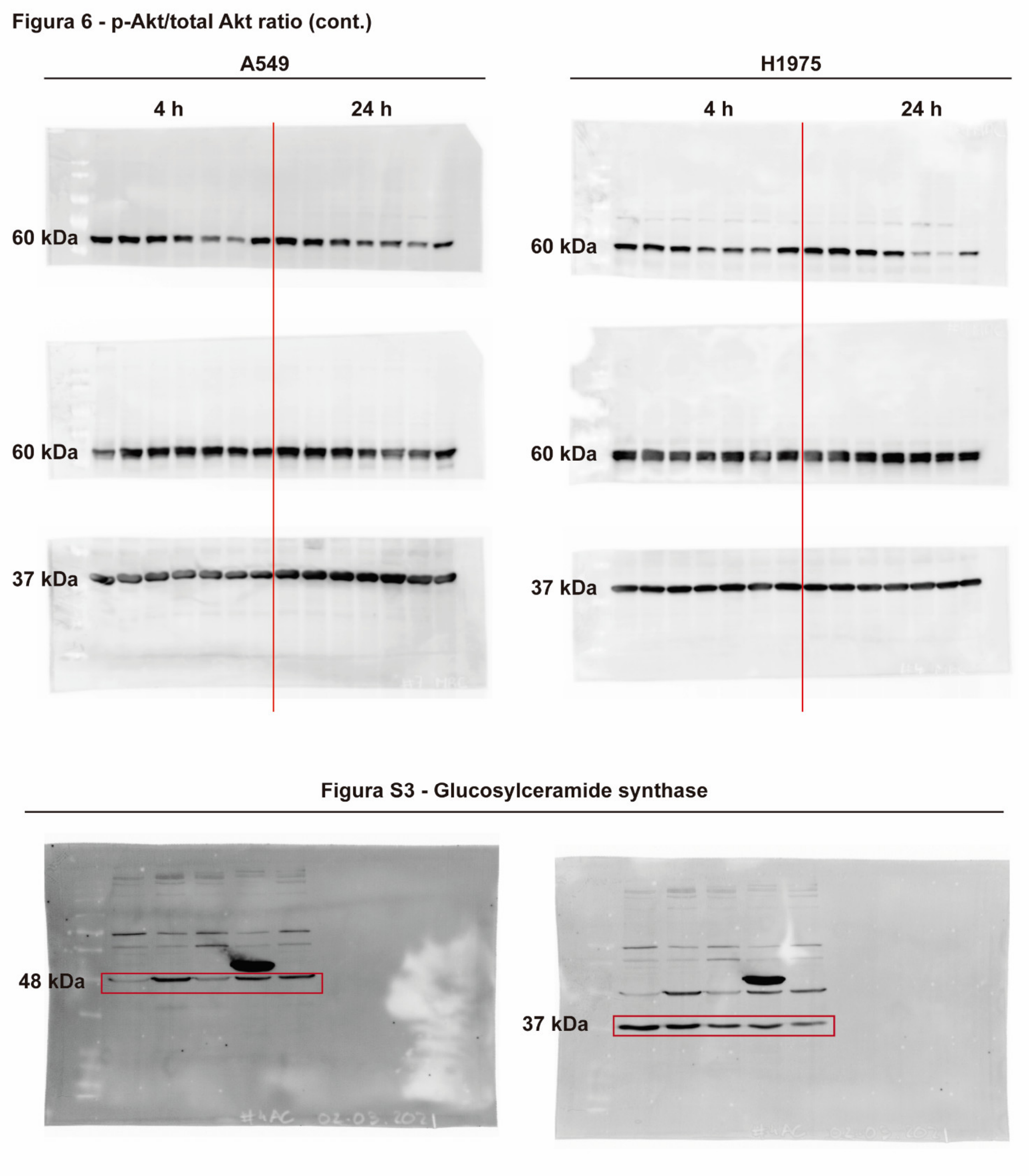

Figure S5. Whole Western Blots. Representative membranes stained with GAPDH (37 kDa); glucosylceramide synthase (48 kDa); β-Tubulin (55 kDa); p-Akt (Ser 473) and Total Akt (60 kDa); lamin B1 (68 kDa) or nucleolin (100 kDa).

Table S1. Multiple Reaction Monitoring (MRM) transitions of the data acquisition method for doxorubicin, C6-ceramide, daunorubicin, and C8-ceramide.

Molecule	Q1 (m/z)	Q3 (m/z)	DP (eV)	CE (eV)	CXP (eV)
Doxorubicin	544.09	396.9	51	17	14
		378.9	51	27	10
		361.0	51	35	10

C6-ceramide	398.48	380.7	60	13	12
		264.5	60	25	20
		81.9	60	57	16
Daunorubicin	528.10	363.3	11	21	4
		321.3	11	39	14
		381.2	11	13	4
C8-ceramide	426.60	408.2	60	15	6
		264.1	60	23	30
		82.4	60	71	6

Compound dependent parameters are described for the Collision energy (CE), collision cell exit potential (CXP), and declustering potential (DP).

Table S2. List of primer nucleotide sequences for qRT-PCR.

Gene	Primer Bank ID	FW/RV	Nucleotide Sequence 5′-3′
AKT1	148806895c1	FW	CCTCCACGACATCGCACTG
		RV	TCACAAAGAGCCCTCCATTATCA
S6K1	55743133c1	FW	CAGTGGGCACCTGTATGCTAT
		RV	ACGAATGGGTGATTTACATCAGC
β-ACTIN	4501885a1	FW	CATGTACGTTGCTATCCAGGC
		RV	CTCCTTAATGTCACGCACGAT
HPRT1	na	FW	TGACACTGGCAAAACAATGCA
		RV	GGTCCTTTTCACCAGCAAGCT

FW—Forward; RV—Reverse; na—not applicable.

Table S3. Cytotoxicity of different liposomal DXR:C6-ceramide combinations against ovarian cancer cell lines.

Drug		IC_{50}						IC_{90}					
		OVCAR-3		TOV-112D		SKOV-3		OVCAR-3		TOV-112D		SKOV-3	
		1 h	4 h	1 h	4 h	4 h	8 h	1 h	4 h	1 h	4 h	4 h	8 h
Liposomal Doxorubicin	L-D	2.75	1.38	5.00	3.17	15.28	6.52	13.52	7.55	36.94	22.80	>50	>50
	[NS]L-D	2.74	1.88	5.68	2.77	14.55	7.36	11.74	10.95	37.52	28.01	>50	>50
	[F3]L-D	1.36	0.28	2.22	0.83	1.74	0.83	4.70	2.58	18.70	7.72	>50	>50
Liposomal Combinations	L-$DC_6$11	1.54	0.93	4.10	1.62	10.03	2.95	7.27	7.06	26.83	16.50	>50	>50
	[NS]L-$DC_6$11	1.53	1.11	4.55	1.55	7.13	2.92	5.12	8.03	27.06	17.65	>50	>50
	[F3]L-$DC_6$11	0.45	0.21	1.43	0.54	0.61	0.44	2.18	1.11	11.93	7.73	>50	22.61
	L-$DC_6$12	1.60	0.97	4.06	1.53	12.10	3.14	7.99	9.34	31.27	14.01	>50	>50
	[NS]L-$DC_6$12	1.98	0.86	3.73	1.59	9.83	2.28	8.38	9.47	30.37	14.74	>50	>50
	[F3]L-$DC_6$12	0.55	0.26	1.34	0.55	2.07	0.43	2.39	1.77	13.31	6.75	>50	16.69

Data represent the IC_{50} and the IC_{90} ([DXR] μM) of the mean dose–response curves calculated through linear interpolation. The indicated ovarian cancer cells were incubated for 1, 4 or 8 h with F3 peptide-targeted liposomal DXR ([F3]L-D) or DXR:C6-ceramide combination at a molar ratio of 1:1 or 1:2 ([F3]L-$DC_6$11 and [F3]L-$DC_6$12, respectively), at DXR serially diluted concentrations. The experiment was further prolonged for total of 96 (TOV-112D), 120 (SKOV-3) or 144 h (OVCAR-3) after which cytotoxicity was assessed. Additional controls included liposomes either functionalized by a non-specific peptide ([NS]L-D, [NS]L-$DC_6$11 and [NS]L-$DC_6$12) or non-targeted (L-D, L-$DC_6$11 and L-$DC_6$12), incubated under the same experimental conditions.

Table S4. Cytotoxicity of different liposomal DXR:C6-ceramide combinations against non-small cell lung cancer cell lines.

**Drug**		**IC_{50}**				**IC_{90}**			
		**H1975**		**A549**		**H1975**		**A549**	
		1 h	4 h	1 h	4 h	1 h	4 h	4 h	8 h
Liposomal Doxorubicin	L-D	3.9	2.5	7.0	4.0	>12.5	>12.5	>50	>50
	[F3]L-D	0.5	0.2	1.0	0.4	5.1	2.1	>50	11.3
Liposomal Combinations	L-$DC_6$11	4.5	3.8	48.8	6.2	>12.5	>12.5	>50	>50
	[F3]L-$DC_6$11	0.7	0.6	2.3	0.4	6.8	3.4	>50	24.6
	L-$DC_6$12	2.9	1.8	8.4	6.1	>12.5	7.6	>50	>50
	[F3]L-$DC_6$12	0.8	0.3	1.1	0.4	6.1	2.3	>50	27.0

Data represent the IC_{50} and the IC_{90} ([DXR] μM) of the mean dose–response curves calculated through linear interpolation of the dose values immediately below or above the 50% and 90% effect, respectively. H1975 and A549 non-small cell lung cancer cells were incubated for 1 or 4 h with F3 peptide-targeted liposomal DXR ([F3]L-D) or DXR:C6-ceramide combination at a molar ratio of 1:1 or 1:2 ([F3]L-$DC_6$11 and [F3]L-$DC_6$12, respectively), at DXR serially diluted concentrations. The experiment was further prolonged for total of 96 (A549) or 120 h (H1975) after which cell death was assessed by the resazurin reduction assay. Additional controls included non-targeted liposomes (L-D, L-$DC_6$11 and L-$DC_6$12), incubated under the same experimental conditions.
